# Low Bandgap Donor-Acceptor π-Conjugated Polymers From Diarylcyclopentadienone-Fused Naphthalimides

**DOI:** 10.3389/fchem.2019.00362

**Published:** 2019-05-29

**Authors:** Xiaolin Li, Jing Guo, Longfei Yang, Minghao Chao, Liping Zheng, Zhongyun Ma, Yuanyuan Hu, Yan Zhao, Huajie Chen, Yunqi Liu

**Affiliations:** ^1^Key Laboratory for Green Organic Synthesis and Application of Hunan Province, and Key Laboratory of Environmentally Friendly Chemistry and Applications of Ministry of Education, College of Chemistry, Xiangtan University, Xiangtan, China; ^2^Key Laboratory for Micro/Nano Optoelectronic Devices of Ministry of Education & Hunan Provincial Key Laboratory of Low-Dimensional Structural Physics and Devices, School of Physics and Electronics, Hunan University, Changsha, China; ^3^Department of Materials Science, Institute of Molecular Materials and Devices, Fudan University, Shanghai, China

**Keywords:** diarylcyclopentadienone-fused naphthalimides, D-A conjugated polymers, optical band gap, electron-transporting materials, charge carrier transport

## Abstract

Two novel aromatic imides, diarylcyclopentadienone-fused naphthalimides (BCPONI-2Br and TCPONI-2Br), are designed and synthesized by condensation coupling cyclopentadienone derivatives at the lateral position of naphthalimide skeleton. It has been found that BCPONI-2Br and TCPONI-2Br are highly electron-withdrawing acceptor moieties, which possess broad absorption bands and very low-lying LUMO energy levels, as low as −4.02 eV. On the basis of both building blocks, six low bandgap D-A copolymers (P1–P6) are prepared via Suzuki or Stille coupling reactions. The optical and electrochemical properties of the polymers are fine-tuned by the variations of donors (carbazole, benzodithiophene, and dithienopyrrole) and π-conjugation linkers (thiophene and benzene). All polymers exhibit several attractive photophysical and electrochemical properties, i.e., broad near-infrared (NIR) absorption, deep-lying LUMO levels (between −3.88 and −3.76 eV), and a very small optical bandgap $\left(E_{\text{g}}^{\text{opt}}\right)$ as low as 0.81 eV, which represents the first aromatic diimide-based polymer with an $E_{\text{g}}^{\text{opt}}$ of <1.0 eV. An investigation of charge carrier transport properties shows that P5 exhibits a moderately high hole mobility of 0.02 cm^2^ V^−1^ s^−1^ in bottom-gate field-effect transistors (FETs) and a typical ambipolar transport behavior in top-gate FETs. The findings suggest that BCPONI-2Br, TCPONI-2Br, and the other similar acceptor units are promising building blocks for novel organic semiconductors with outstanding NIR activity, high electron affinity, and low bandgap, which can be extended to various next-generation optoelectronic devices.

## Introduction

Soluble donor–acceptor (D–A) conjugated polymers can offer a flexible and tunable electronic structure and optoelectronic properties (Guo et al., 2014; Dou et al., 2015), which encourage the incessant exploration of multiple potential applications in next-generation optoelectronic devices, including organic light-emitting diodes (OLEDs) (Grimsdale et al., 2009), organic photovoltaics (OPVs) (Cheng et al., 2009), organic field-effect transistors (OFETs) (Hu et al., 2018; Yang et al., 2018), and organic photodetectors (OPDs) (Gong et al., 2009). By independently selecting or modifying D/A segments, one can readily regulate optical properties, electronic structures (bandgap and HOMO/ LUMO energy levels), and charge carrier transport of the target D-A polymers (Hwang et al., 2012; Cui and Wudl, 2013; Zhao et al., 2015; Chen et al., 2016; Fei et al., 2016; Li et al., 2016). Such a D-A strategy has led to the rapid development of numerous D-A conjugated polymers and makes a great contribution to promote device performance in organic electronics (Guo et al., 2014; Dou et al., 2015; Yang et al., 2018). In recent years, some classical organic dyes, such as diketopyrrolopyrrole (DPP) (Zou et al., 2009; Li et al., 2011, 2013c) and isoindigo (IDG) (Stalder et al., 2010; Lei et al., 2011; Mei et al., 2011; Gao et al., 2017), have been successfully used as the acceptor building blocks to construct low bandgap D-A conjugated polymers for various optoelectronic devices, especially in OPVs and OFETs. Hole or electron mobilities higher than 5.0 cm^2^ V^−1^ s^−1^ (Chen et al., 2012; Gao et al., 2015) and a power conversion efficiency (PCE) of above 8.0% (Hendriks et al., 2013) have been reported for the DPP-containing D-A polymers.

**Figure 1:**
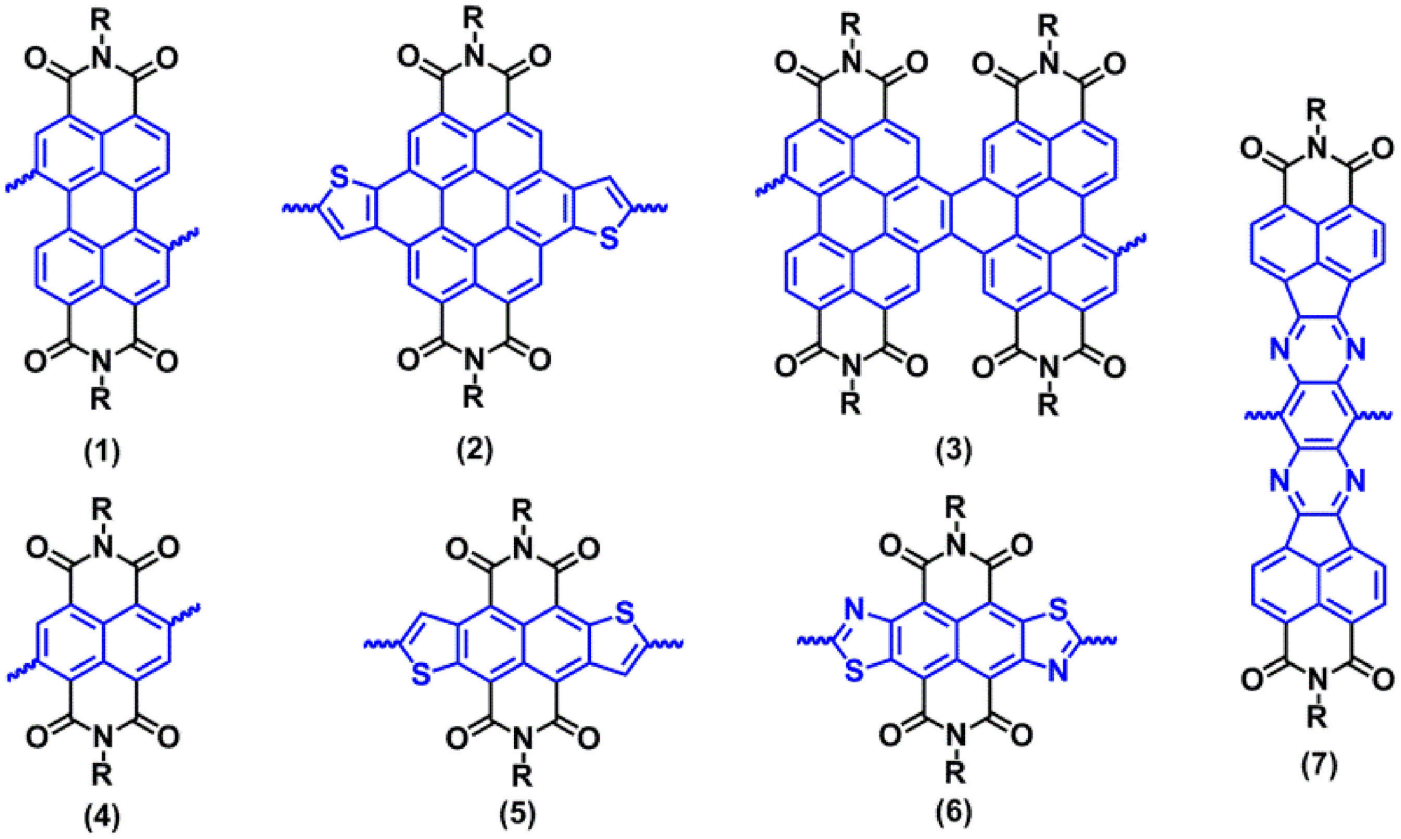
Some perylene diimide and naphthalene diimide building blocks in conjugated polymers.

In view of the urgent need for electron-transporting materials, aromatic imides like rylene diimides have been widely studied (Zhan et al., 2011). Moreover, they have also become attractive acceptor building blocks for *n*-type conjugated polymers due to the strong electron-deficient feature, high electron mobility, tunable solubility supported by *N*-alkylation, and excellent chemical and photochemical stability (Zhan et al., 2011). So far, the most studied rylene diimides are perylene diimide (PDI, **1**) and naphthalene diimide (NDI, **4**) (Figure 1). Zhan and co-workers reported the synthesis of the first soluble PDI-dithienothiophene copolymers (Zhan et al., 2007), which yielded a moderately high electron mobility of 0.013 cm^2^ V^−1^ s^−1^ and a PCE value of 1.5% when used as the active layers in top-gate OFETs and all-polymer OPVs, respectively. Since then, great efforts have been made to structurally modify PDI acceptor units, thereby generating a sets of core-extended PDI analogs (Choi et al., 2011; Usta et al., 2012; Cai et al., 2017, 2018), such as dithienocoronene diimides (DTCDI, **2**) (Choi et al., 2011; Zhou et al., 2012) and naphthodiperylenetetraimide (NTDPI, **3**) (Guo et al., 2017) (Figure 1). Compared with PDIs, both DTCDI and NTDPI acceptor building blocks possess larger conjugation backbones than PDI, which could facilitate strong intermolecular interactions and charge carriers transport of the polymers (Zhou et al., 2012). Facchetti and co-workers reported the synthesis of soluble D-A polymers containing DTCDI and thiophene units (Usta et al., 2012), which exhibited good hole and electron mobilities of 0.04 and 0.3 cm^2^ V^−1^ s^−1^, respectively. Zhao and co-workers synthesized the NTDPI and vinylene-linked *n*-type polymer and afforded an excellent PCE value of 8.59% in the inverted all-polymer OPVs (Guo et al., 2017).

Naphthalene diimide (NDI, **4**, Figure 1) is the other strongly electron-deficient building block for the development of polymer electron-transporting materials. Watson and co-workers. pioneered the use of NDI as an acceptor building block in D-A copolymers with a tunable optical bandgap ($E_{\text{g}}^{\text{opt}}$) ranging from 1.7 to 1.1 eV (Guo and Watson, 2008). Later, Facchetti and co-workers reported the synthesis and OFETs properties of an NDI-bithiophene polymer (N2200), which exhibited an impressive electron mobility up to 0.85 cm^2^ V^−1^ s^−1^ under ambient conditions (Yan et al., 2009). Thanks to structural optimization and device engineering, copolymers-based NDI units have also very recently provided near state-of-the-art electron mobilities >7.0 cm^2^ V^−1^ s^−1^ (Zhao et al., 2017; Wang et al., 2019). An all-polymer OPV device, reported by Huang and co-workers has been further developed to a benchmark PCE value of 11% (Li et al., 2019), which fabricated from an N2200 acceptor and polymer donor (PTzBI-Si). Recently, a core-extended strategy has been successfully utilized to prepare various heteroaromatic-fused NDI derivatives, such as thiophene-fused NDI (NDTI, **5**) (Fukutomi et al., 2013), thiazole-fused NDI (NDTZ, **6**) (Chen et al., 2013), and pyrazine-fused NDI (BFI, **7**) (Figure 1) (Li et al., 2013b). These core-extended NDI units can afford a rigid π-conjugation backbone with distinct electronic structures as relative-to-simple core-linked NDIs, which has been developed as the promising building blocks for polymer electron-transporting materials (Chen et al., 2013; Fukutomi et al., 2013; Li et al., 2013a). A typical example of the core-extended NDI is tetraazabenzodifluoranthene diimide (BFI, **7**) reported by Jenekhe and co-workers; moreover, it was found that a BFI-containing copolymer has a large lateral extension (2.0 nm) of π-conjugation and perfect lamellar ordering, thereby achieving high electron mobilities of 0.3 cm^2^ V^−1^ s^−1^ (Li et al., 2013a).

**Figure 2:**
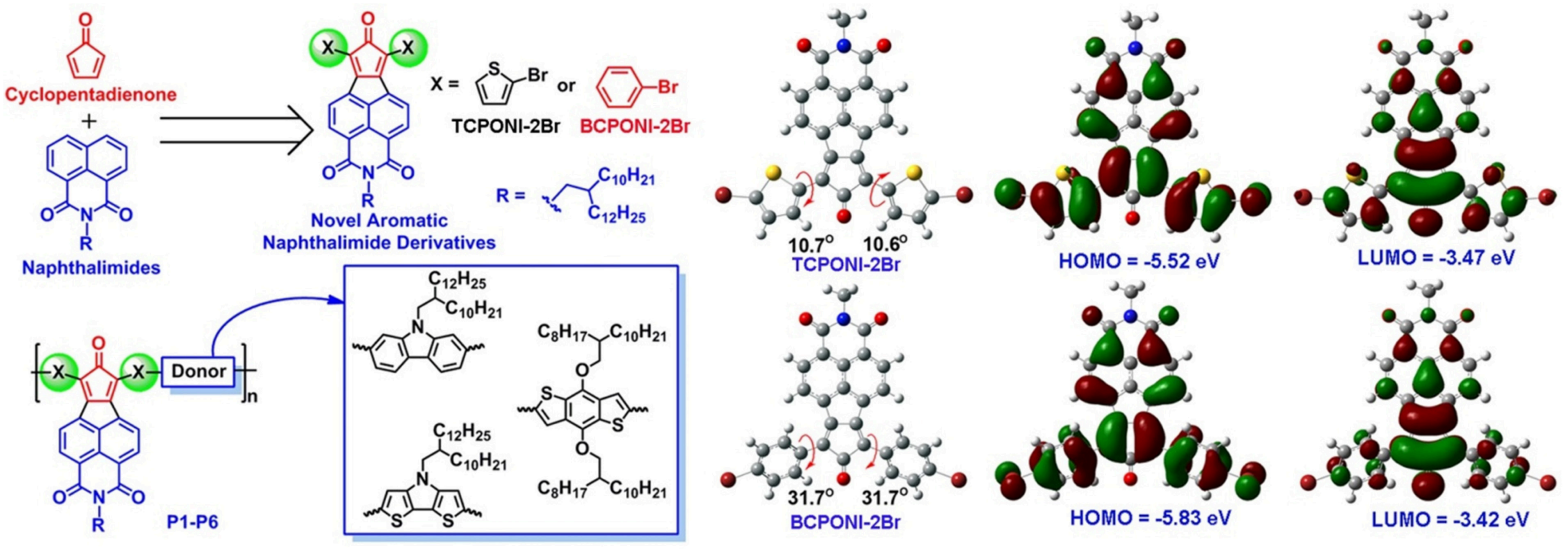
Molecular design and chemical structures of the two monomers (BCPONI-2Br and TCPONI-2Br) and their D-A copolymers (P1–P6). The optimized structures, molecular orbitals, and HOMO/LUMO energy levels of the two monomers as obtained from density functional theory (DFT) calculations.

Diarylcyclopentadienone-fused naphthalimide (CPONI, Figure 2) is a novel family of aromatic imide building blocks that originally derived from naphthalimide and cyclopentadienone units and is similar in structure to cyclopentadieneones. Wudl and co-workers reported the synthesis and OFET properties of cyclopentadieneones-containing oligomers (Walker et al., 2008; Yang et al., 2008), which demonstrated very small bandgaps as low as 0.9 eV and a moderate hole mobility of 2.26 × 10^−2^ cm^2^ V^−1^ s^−1^ (Yang et al., 2008). Compared with cyclopentadieneone analogs, the combined advantages of both NDI and cyclopentadienone units endow CPONI acceptors with extended π-conjugation backbone, enhanced electron-withdrawing capacity, as well as tunable solubility that supported by *N*-alkylation. The imide nitrogens in the CPONI units allow attachment of solublizing side chains in the polymer backbone in order to tune solubility and self-organization without disrupting backbone's coplanarity. Thus, CPONI derivatives are of great interest to construct low band-gap D-A conjugated polymers with promising electronic structure and optoelectronic properties. Nevertheless, literature reports on the synthesis, reactivity, and optoelectronic properties of diarylcyclopentadienone-fused naphthalimide derivatives are rarely seen (Ding et al., 2015; Ishikawa et al., 2018). To our knowledge, the CPONI-based polymers have not been reported.

In this article, two novel CPONI-derived acceptor building-blocks, diphenylcyclopentadienone- fused naphthalimide (BCPONI-2Br) and dithienylcyclopentadienone-fused naphthalimide (TCPONI-2Br) (Figure 2), were designed and synthesized for low bandgap D-A polymers. Herein, thiophene and benzene rings were selected as the π-linker units of TCPONI-2Br and BCPONI-2Br, respectively, in order to manipulate backbone coplanarity, energy levels, and absorption of the target polymers. As revealed by theoretical calculations, the dihedral angles of thiophene-flanked TCPONI-2Br (ca. 10.6 and 10.7°) are smaller than those of benzene-flanked BCPONI-2Br (ca. 31.7°), indicating better backbone coplanarity for TCPONI-2Br. Interestingly, the attachment of electron-rich thiophene units endows TCPONI-2Br with a slightly reduced LUMO value (ca. −3.47 eV) but sharply improved the HOMO value (ca. −5.52 eV) relative to BCPONI-2Br. Such deep-lying LUMO values indicate that TCPONI-2Br (ca. −3.47 eV) and BCPONI-2Br (ca. −3.42 eV) are promising strong acceptor units for electron-transporting polymers. By using both building blocks as the electron acceptors, six novel D-A conjugated polymers with different electron-donating capability donor units (carbazole, benzodithiophene, and dithienopyrrole) were prepared by Suzuki or Stille coupling reactions. The polymers exhibit very attractive photophysical and electrochemical properties, i.e., broad near-infrared (NIR) absorption extended to 1,600 nm and adjustable $E_{\text{g}}^{\text{opt}}$ values from 0.81 to 1.55 eV, which was realized by employing different donor units and π-conjugation linkers. To our satisfaction, an ultralow $E_{\text{g}}^{\text{opt}}$ of 0.81 eV was achieved for TCPONI-containing polymer (P6), which represents the first aromatic diimide-based polymer with the $E_{\text{g}}^{\text{opt}}$ <1.0 eV.

## Experimental Section

### General Measurements

Nuclear magnetic resonance spectra (^1^H NMR and ^13^C NMR) were collected on a Bruker AVANCE 400 spectrometer. Mass spectrometry (MALDI-TOF-MS) was performed on a Bruker AutoflexTM III instrument. Molecular weight was determined by high temperature gel permeation chromatography (150°C in 1,2,4-trichlorobenzene) on a Polymer Labs PL 220 system. UV–vis–NIR absorption spectroscopy was measured using a Perkin-Elmer Lamada 750 UV/vis spectrometer. Thermogravimetric analysis (TGA) was recorded on a Perkin-Elmer TGA-7 Analyzer with a heating rate of 10°C min^−1^. Differential scanning calorimetry (DSC) was measured on a DSC Q10 instrument with the heating/cooling rates of 10°C min^−1^. Cyclic voltammetry (CV) was performed on an electrochemistry workstation (CHI660E, Chenhua Shanghai) using a three-electrode cell. For the characterization of small molecules, three-electrode cell with a Pt wire counter electrode, a Ag/AgCl (KCl, Sat'd) reference electrode, and a glassy carbon working electrode was utilized. For the measurement of polymers, a Pt wire, a Ag/AgCl (KCl, Sat'd) electrode and a Pt disk drop-coated with polymer film were used as the counter, reference electrode, and working electrode, respectively. The electrolytes were anhydrous and N_2_-saturated tetrabutylammonium hexafluorophosphate (TBAPF6, 0.1 M) solutions in dichloromethane or acetonitrile. A Fourier-transform infrared spectroscopy (FT-IR) was carried out on a Nicolet 6700 FT-IR spectrometer in a scan range from 4,000 to 600 cm^−1^. The film surface morphology was characterized by atomic force microscopy (AFM, Bruker Multi-Mode 8 microscope) using a tapping mode. Grazing incidence X-ray diffraction (GIXRD) experiments were performed to characterize film organization. The polymer film samples were illuminated at a constant incidence angle of 0.2°.

### Materials and Synthesis

Tetrahydrofuran (THF) and chlorobenzene were dried and distilled prior to use. All the reagents and chemicals were purchased from Chem Greatwall, Derthon, and Alfa Aesar. Some important intermediates, including 1,3-dithiophenyl-2-propanone (1) (Walker et al., 2008), 1,3-bis(4-bromophenyl)-2-propanone (3) (Walker et al., 2008), 2-(2-decyltetradecyl)-1*H*-indeno[6,7,1-*def*]isoquinoline-1,3,6,7(2*H*)-tetraone (4) (Li et al., 2013b), *N*-(2-decyltetradecyl)-2,7-bis-(4,4,5,5-tetramethyl-1,3,2-dioxaborolane-2-yl)carbazole (Kim et al., 2011), 2,6-bis(trimethyltin)-4,8-di(2-hexyl)decyloxybenzo[1,2-*b*;3,4-*b*′]dithiophene (Mei et al., 2013), and 2,6-bis(trimethylstannyl)-*N*-(2-decyltetradecyl)dithieno[3,2-*b*:2′,3′-*d*]pyrrole (Zhang et al., 2010), were synthesized according to literature procedures, respectively.

### Synthesis of Compound 2

Under nitrogen, a mixture of 1,3-dithiophenyl-2-propanone (1.2 g, 5.40 mmol) and CHCl_3_ (25 mL) was stirred at 0°C. Next, 2.11 g of *N*-bromobutanimide (NBS) (11.88 mmol) was slowly added to the reaction mixture. After stirring for 5 h at room temperature, the reaction was quenched with water. The organic layer was extracted by CH_2_Cl_2_ and dried over anhydrous MgSO_4_. After removal of the solvent, the crude product was purified by column chromatography on silica gel using a mixed eluent of petroleum ether and CH_2_Cl_2_ (3:1, v/v) to afford a yellow solid (1.05 g, 51%). ^1^H NMR (400 MHz, CDCl_3_), δ (ppm): 6.93–6.92 (d, *J* = 3.7 Hz, 2H), 6.65–6.64 (d, *J* = 3.7 Hz, 2H), 3.91 (s, 4H). ^13^C NMR (100 MHz, CDCl_3_), δ (ppm): 201.47, 135.94, 129.78, 127.53, 111.81, 42.74. FT-IR spectra data: υ_C = O_: 1,698 and 1,663 cm^−1^.

### Synthesis of BCPONI-2Br

Under nitrogen, to a mixture of compound 4 (500 mg, 0.85 mmol), 1,3-bis(4-bromophenyl)-2-propanone (313 mg, 0.85 mmol), and ethanol (25 mL), 24 mg of KOH (0.42 mmol) in 5 mL of ethanol were added slowly. The solution changed to red immediately and then precipitates formed gradually. The mixture was refluxed for 30 min and then cooled down to room temperature. The organic layer was extracted by CH_2_Cl_2_ and dried with anhydrous MgSO_4_. After removal of the solvent, the crude product was purified by column chromatography on silica gel using a mixed eluent of petroleum ether and CH_2_Cl_2_ (1:2, v/v) to yield a brown solid (470 mg, 60%). ^1^H NMR (400 MHz, CDCl_3_), δ (ppm): 8.60–8.58 (d, *J* = 7.6 Hz, 2H), 8.22–8.20 (d, *J* = 7.6 Hz, 2H), 7.73 (br, 8H), 4.13–4.12 (d, *J* = 4.0 Hz, 2H), 1.98 (br, 1H), 1.21 (m, 40H), 0.86 (m, 6H). ^13^C NMR (100 MHz, CDCl_3_), δ (ppm): 199.52, 163.35, 151.73, 142.55, 135.34, 132.61, 132.23, 130.53, 128.95, 126.73, 124.58, 124.08, 122.56, 121.34, 44.76, 36.67, 31.95, 31.77, 30.07, 29.69, 29.38, 26.54, 22.71, 14.14. FT-IR spectra data: υ_C = O_: 1,698 and 1,663 cm^−1^. HRMS (MALDI–TOF): m/z [M]^+^ calcd for (C_53_H_62_Br_2_NO_3_): 918.3090; found: 918.3090.

### Synthesis of TCPONI-2Br

Under nitrogen, to a mixture of compound 4 (500 mg, 0.85 mmol), 1,3-bis(5-bromothiophenyl)-2-propanone (324 mg, 0.85 mmol), and ethanol (25 mL), 24 mg of KOH (0.42 mmol) in 5 mL of ethanol were added slowly. The red mixture was then refluxed for 30 min. The organic layer was extracted by CH_2_Cl_2_ and dried with anhydrous MgSO_4_. After filtration and removal of the solvent, the crude product was purified by column chromatography on silica gel using a mixed eluent of petroleum ether and CH_2_Cl_2_ (1:2, v/v) to yield a brown solid (476 mg, 60%). ^1^H NMR (400 MHz, CDCl_3_), δ (ppm): 8.43–8.41 (d, *J* = 7.6 Hz, 2H), 8.05–8.03 (d, *J* = 7.7 Hz, 2H), 7.37–7.36 (d, *J* = 4.0 Hz, 2H), 6.99–6.98 (d, *J* = 4.0 Hz, 2H), 4.10–4.08 (d, *J* = 7.1 Hz, 2H), 1.98 (br, 1H), 1.38–1.20 (m, 40H), 0.86 (m, 6H). ^13^C NMR (100 MHz, CDCl_3_), δ (ppm): 197.23, 163.02, 146.76, 141.29, 133.36, 133.24, 131.87, 130.60, 130.04, 125.93, 122.08, 121.80, 118.18, 117.01, 36.93, 31.98, 31.74, 30.25, 29.76, 29.73, 29.42, 26.50, 22.73, 14.16. FT-IR spectra data: υ_C = O_: 1,698 and 1,660 cm^−1^. HRMS (MALDI–TOF): m/z [M]^+^ calcd for (C_49_H_58_Br_2_NO_3_S_2_): 930.2219; found: 930.2226.

### Synthesis of P1

Under nitrogen, a mixture of BCPONI-2Br (147 mg, 0.16 mmol), *N*-(2-decyltetradecyl)-2,7-bis-(4,4,5,5-tetramethyl-1,3,2-dioxaborolane-2-yl)carbazole (124 mg, 0.16 mmol), Pd(PPh_3_)_2_Cl_2_ (15 mg), chlorobenzene (5 mL), 2 mL of Na_2_CO_3_ aqueous solution (2M), and a drop of aliquat 336 was added into a 25 mL Schlenk tube. The tube was charged with nitrogen through a freeze-pump-thaw cycle for three times. The mixture was stirred at 100°C for 5 h in the absence of light. After cooling to room temperature, the mixture was dropped into a mixed solution of methanol (200 mL) and concentrated hydrochloric acid (5 mL) and stirred for another 0.5 h. The dark solid was collected and Soxhlet-extracted with ethanol, acetone, hexane, and chlorobenzene. After removal of chlorobenzene, a black solid was obtained (170 mg, 84%). ^1^H NMR (500 MHz, C_2_D_2_Cl_4_, 373 K), δ (ppm): 8.70–7.31 (br, 18H), 4.50–4.00 (br, 4H), 2.31 (br, 1H), 2.12 (br, 1H), 1.60–0.70 (m, 92H). FT-IR spectra data: υ_C = O_: 1,699 and 1,664 cm^−1^. GPC: *M*_n_ = 7.47 kDa, *M*_w_ = 24.12 kDa, PDI = 3.23.

### Synthesis of P2

Under nitrogen, a mixture of BCPONI-2Br (147 mg, 0.16 mmol), 2,6-bis(trimethyltin)-4,8-di(2-hexyl)decyloxybenzo[1,2-*b*;3,4-*b*′]dithiophene (177 mg, 0.16 mmol), Pd_2_(dba)_3_ (9 mg), P(o-tol)_3_ (15 mg), and anhydrous chlorobenzene (5 mL) was added into a 25 mL Schlenk tube. The tube was subsequently charged with nitrogen through a freeze-pump-thaw cycle for three times. The mixture was heated to 120°C and stirred for 60 h in the absence of light. After cooling to room temperature, the mixture was dropped into a mixed solution of methanol (200 mL) and concentrated hydrochloric acid (5 mL) and stirred for another 0.5 h. The black solid was collected and further Soxhlet-extracted with ethanol, acetone, hexane, and chlorobenzene. After removal of chlorobenzene, P2 was obtained as a black solid (227 mg, 92%). ^1^H NMR (500 MHz, C_2_D_2_Cl_4_, 373 K), δ (ppm): 8.70–7.50 (br, 14H), 4.50–4.00 (br, 6H), 2.20–2.00 (m, 3H), 2.00–0.70 (m, 122H). FT-IR spectra data: υ_C = O_: 1,700 and 1,666 cm^−1^. GPC: *M*_n_ = 55.31 kDa, *M*_w_ = 75.67 kDa, PDI = 1.37.

### Synthesis of P3

A mixture of BCPONI-2Br (147 mg, 0.16 mmol), 2,6-bis(trimethylstannyl)-*N*-(2-decyltetradecyl)-dithieno[3,2-*b*:2′,3′-*d*]pyrrole (135 mg, 0.16 mmol), Pd_2_(dba)_3_ (9 mg), P(*o*-tol)_3_ (15 mg), and anhydrous chlorobenzene (5 mL) was added to a 25 mL Schlenk tube. The tube was then charged with nitrogen through a freeze-pump-thaw cycle for three times. The mixture was heated to 115°C and stirred for 72 h under nitrogen atmosphere. After cooling to room temperature, the mixture was dropped into a mixed solution of methanol (200 mL) and concentrated hydrochloric acid (5 mL) and stirred for another 0.5 h. The solid product was collected and Soxhlet-extracted with ethanol, acetone, hexane, and chlorobenzene. After removal of chlorobenzene, a black solid was obtained (188 mg, 92%). ^1^H NMR (500 MHz, C_2_D_2_Cl_4_, 373 K), δ (ppm): 8.70–7.00 (br, 14H), 4.50–3.70 (br, 4H), 2.18–2.04 (br, 2H), 1.70–0.70 (m, 92H). FT-IR spectra data: υ_C = O_: 1,697 and 1,662 cm^−1^. GPC: *M*_n_ = 23.50 kDa, *M*_w_ = 45.82 kDa, PDI = 1.95.

### Synthesis of P4

A mixture of TCPONI-2Br (149 mg, 0.16 mmol), *N*-(2-decyltetradecyl)-2,7-bis-(4,4,5,5-tetramethyl-1,3,2-dioxaborolane-2-yl)carbazole (124 mg, 0.16 mmol), Pd(PPh_3_)_2_Cl_2_ (15 mg), chlorobenzene (5 mL), 2 mL of Na_2_CO_3_ aqueous solution (2 M), and a drop of aliquat 336 was added to a 25 mL Schlenk tube. The tube was then charged with nitrogen through a freeze-pump-thaw cycle for three times. The mixture was stirred at 100°C for 5 h under nitrogen atmosphere. After cooling to room temperature, the mixture was dropped into a mixed solution of methanol (200 mL) and concentrated hydrochloric acid (5 mL) and stirred for another 0.5 h. The crude product was collected and Soxhlet-extracted with ethanol, acetone, hexane, and chlorobenzene. After removal of chlorobenzene, P4 was obtained as a black solid (171 mg, 84%). ^1^H NMR (500 MHz, C_2_D_2_Cl_4_, 373 K), δ (ppm): 9.00–6.50 (br, 14H), 4.50–3.80 (br, 4H), 2.00–0.60 (m, 94H). FT-IR spectra data: υ_C = O_: 1,697 and 1,660 cm^−1^. GPC: *M*_n_ = 20.10 kDa, *M*_w_ = 40.41 kDa, PDI = 2.01.

### Synthesis of P5

A mixture of TCPONI-2Br (149 mg, 0.16 mmol), 2,6-bis(trimethyltin)-4,8-di(2-hexyl)decyloxybenzo[1,2-*b*;3,4-*b*′]dithiophene (177 mg, 0.16 mmol), Pd_2_(dba)_3_ (9 mg), P(o-tol)_3_ (15 mg), and anhydrous chlorobenzene (5 mL) was added to a 25 mL Schlenk tube. The mixture was charged with nitrogen through a freeze-pump-thaw cycle for three times and then stirred at 110°C for 3 h in the absence of light. After cooling to room temperature, the mixture was dropped into a mixed solution of methanol (200 mL) and concentrated hydrochloric acid (5 mL) and stirred for another 0.5 h. The solid product was collected and Soxhlet-extracted with ethanol, acetone, hexane, and chlorobenzene. After removal of chlorobenzene, a black solid was obtained (239 mg, 96%). ^1^H NMR (500 MHz, C_2_D_2_Cl_4_, 373 K), δ (ppm): 9.00–6.50 (br, 10H), 4.60–3.80 (br, 6H), 2.50–0.60 (m, 125H). FT-IR spectra data: υ_C = O_: 1,698 and 1,662 cm^−1^. GPC: *M*_n_ = 32.21 kDa, *M*_w_ = 77.10 kDa, PDI = 2.40.

### Synthesis of P6

A mixture of TCPONI-2Br (149 mg, 0.16 mmol), 2,6-bis(trimethylstannyl)-*N*-(2-decyltetradecyl)-dithieno[3,2-*b*:2′,3′-*d*]pyrrole (135 mg, 0.16 mmol), Pd_2_(dba)_3_ (9 mg), P(*o*-tol)_3_ (15 mg), and anhydrous chlorobenzene (5 mL) was added to a 25 mL Schlenk tube. The mixture was charged with nitrogen through a freeze-pump-thaw cycle for three times and then stirred at 115°C for 24 h in the absence of light. After cooling to room temperature, the mixture was dropped into a mixed solution of methanol (200 mL) and concentrated hydrochloric acid (5 mL) and stirred for another 0.5 h. The solid product was collected and Soxhlet-extracted with ethanol, acetone, hexane, and chlorobenzene. After removal of chlorobenzene, a black solid was obtained (196 mg, 95%). ^1^H NMR (500 MHz, C_2_D_2_Cl_4_, 373 K), δ (ppm): 8.00–6.40 (br, 10H), 4.50–3.60 (br, 4H), 2.40–0.50 (m, 94H). FT-IR spectra data: υ_C = O_: 1,696 and 1,660 cm^−1^. GPC: *M*_n_ = 7.06 kDa, *M*_w_ = 16.49 kDa, PDI = 2.34.

## Results and Discussion

### Synthesis

**Figure 3:**
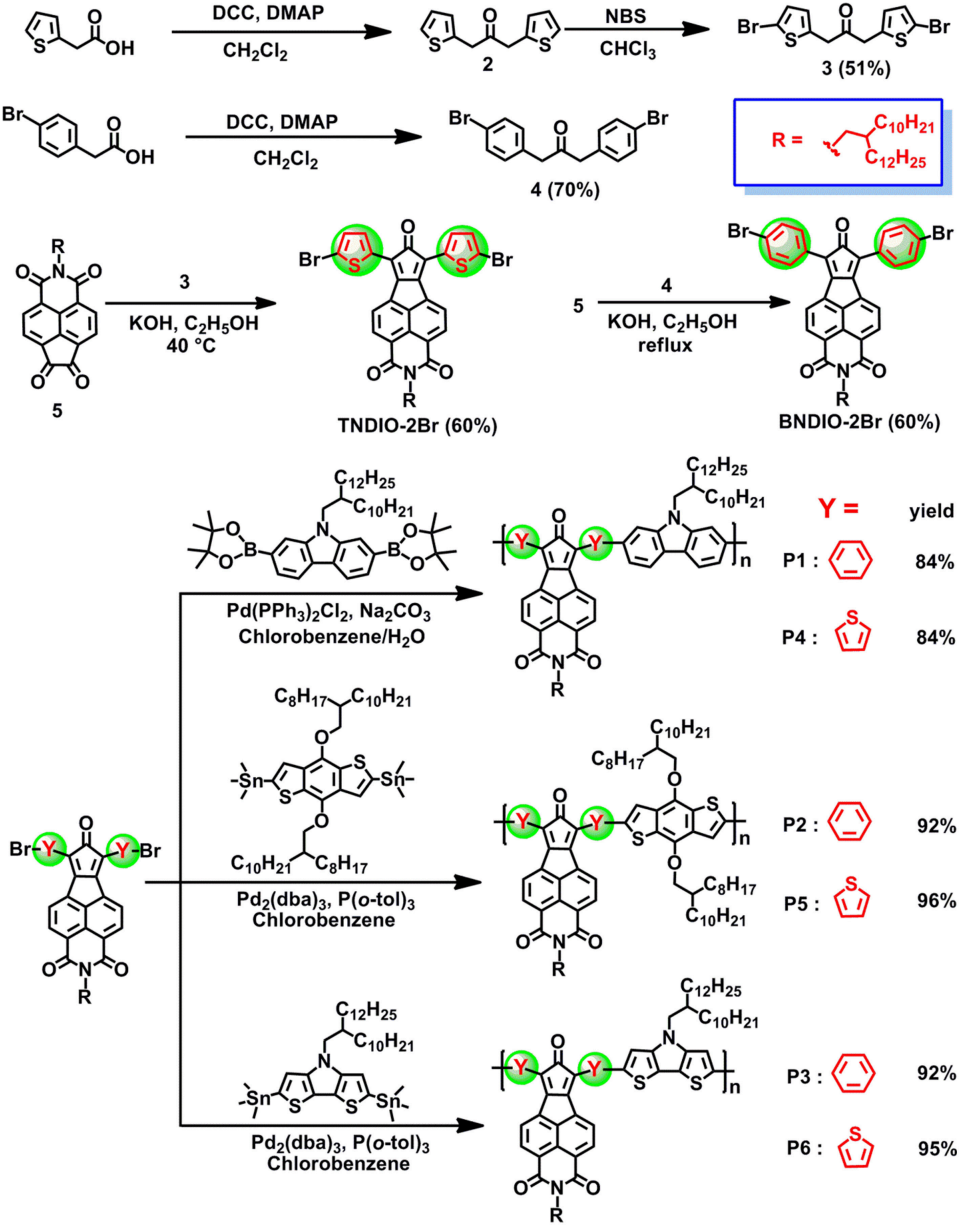
Synthetic route for the CPONI-based monomers and their copolymers.

Figure 3 describes the synthetic routes of the two CPONI-containing monomers and their D–A copolymers. Two important intermediates, 1,3-dithiophenyl-2-propanone (1) (Walker et al., 2008) and 1,3-bis(4-bromophenyl)-2-propanone (3) (Walker et al., 2008), were synthesized according to the reported procedures, respectively. The synthesis of both monomers started from the self-condensation reactions of thiopheneacetic acid or 4-bromobenzeneacetic acid to give compounds 1 and 3, respectively. Then, bromination of compound 1 with NBS afforded 1,3-bis(5-bromothiophenyl)-2-propanone (2) in 51% yields. Finally, double Knoevenagel condensation reactions were readily performed between diketone-containing 5 and compounds 1 or 3 to produce two black solids, BCPONI-2Br and TCPONI-2Br, respectively. The chemical structures of all the intermediates and dibrominated monomers were confirmed by ^1^H NMR and ^13^C NMR (Figures S11–S16). In addition, the diboronicester reagent of carbazole (Kim et al., 2011), as well as distannyl derivatives of benzodithiophene (Mei et al., 2013) and dithienopyrrole (Zhang et al., 2010), were prepared according to similar literature procedures, and their ^1^H NMR data are provided in Figures S17–S19.

The target copolymers were synthesized via the standard palladium-catalyzed Suzuki or Stille coupling reaction between the dibrominated monomers (BCPONI-2Br and TCPONI-2Br) and electron-donated monomers (carbazole, benzodithiophene, and dithienopyrrole). For benzodithiophene- and dithienopyrrole-containing polymers (P2, P3, P5, and P6), Stille polymerization was conducted using Pd_2_(dba)_3_/P(*o*-tol)_3_ as the catalyst, while P1 and P4 was synthesized by Pd(PPh)_2_Cl_2_-catalyzed Suzuki polymerization. In fact, when we chose Pd_2_(dba)_3_/P(*o*-tol)_3_ as the catalyst, all the resulted P1 and P4 samples were insoluble due to “over-polymerization.” Fortunately, we obtained all the solution-processable polymer samples (P1–P6) that can be dissolved in warm organic solvents (chloroform, chlorobenzene, and xylene).

**Figure 4:**
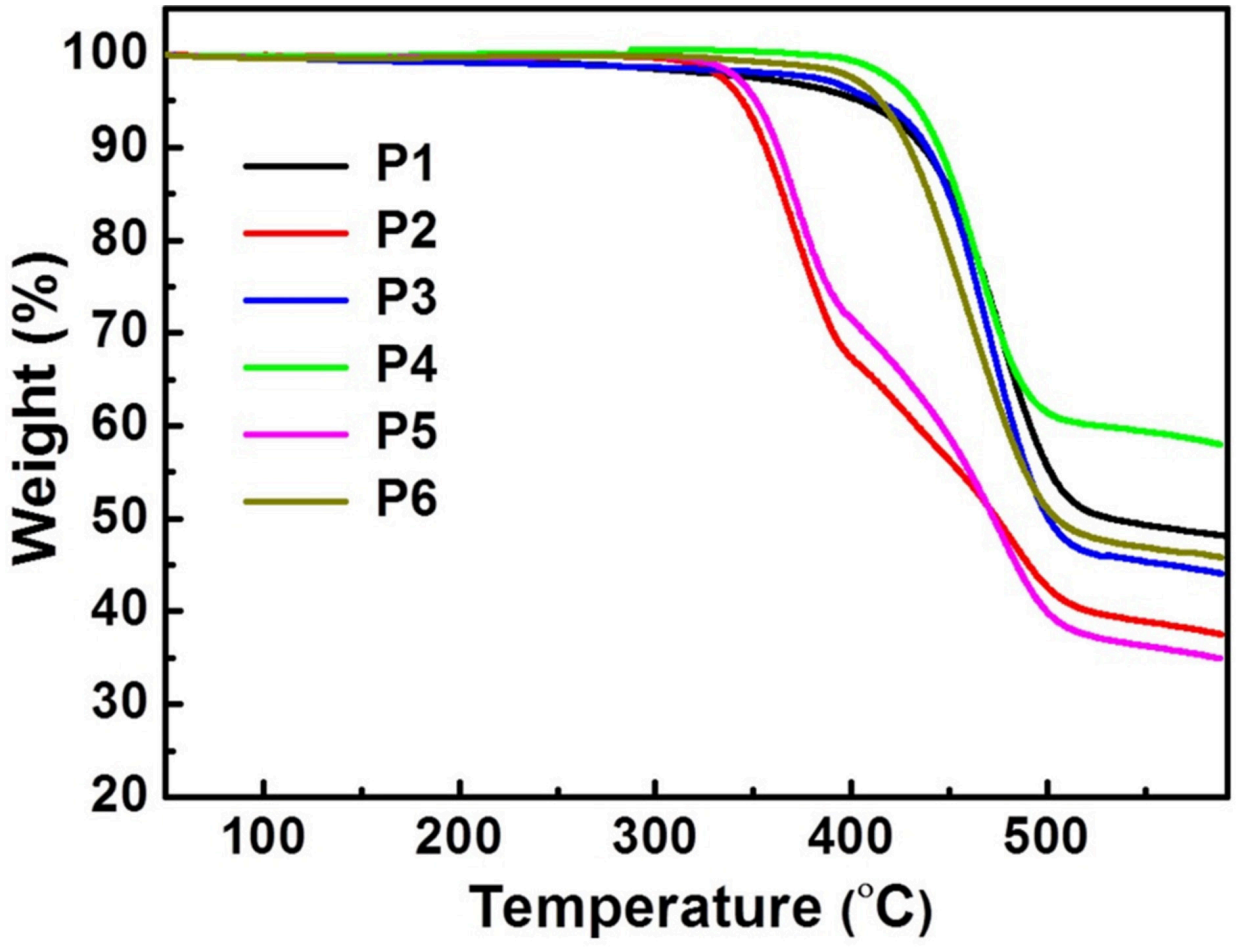
TGA curves of the CPONI-based polymers.

Molecular weight of the polymers was measured by high-temperature (150°C) GPC and calibrated by monodisperse polystyrene. The observed number-average molecular weights (*M*_n_) are 7.06–55.31 kDa, and the polydispersity indices are 1.37–3.23 (Table 1 and Figures S1–S6). The thermal properties of the polymers were investigated by TGA and DSC instruments under nitrogen. As seen from Figure 4, all the polymers display excellent thermal stability. For the carbazole- and dithienopyrrole-containing polymers (P1, P3, P4, and P6), the thermal decomposition temperature (*T*_d_) at 5% weight loss are above 398°C, while the benzodithiophene-containing P2 and P5 exhibit much lower *T*_d_ (ca. 350°C). Additionally, no obvious phase transition was detected from the DSC measurements during the heating/cooling scan between room temperature and 280°C (Figure S7). FT-IR spectroscopy of the polymers displays typical characteristic bands (υ_C = O_) at ca. 1,697 and 1,660 cm^−1^, thereby providing direct evidence for the carbonyl groups in the polymers (Figures S8, S9). Although ^1^H NMR spectroscopy of the polymers were collected at a high temperature of 373 K, only broad and featureless signals were detected at aromatic (δ = 7.0–9.0 ppm) and alkyl bands (δ = 3.5–4.5 and 0.5–2.5 ppm) (Figures S20–S25). The results suggest that a common phenomenon, i.e., strong interchain aggregation (Guo and Watson, 2011), also exists in the newly-developed CPONI-containing polymers and cannot be broken even at high temperature of 373 K.

**Table 1:** Molecular weight, yield, and decomposition temperature of the polymers.

**Polymer**	**Yield**	***M*_**n**_**	***M*_**w**_**	**PDI**	***T*_**d**_**
	**(%)**	**(kDa)**	**(kDa)**		**(^**°**^C)**
P1	84	7.47	24.12	3.23	405
P2	92	55.31	75.67	1.37	344
P3	92	23.50	45.82	1.95	412
P4	84	20.10	40.41	2.01	429
P5	96	32.21	77.10	2.40	351
P6	95	7.06	16.49	2.34	413

### Optical Properties

**Figure 5:**
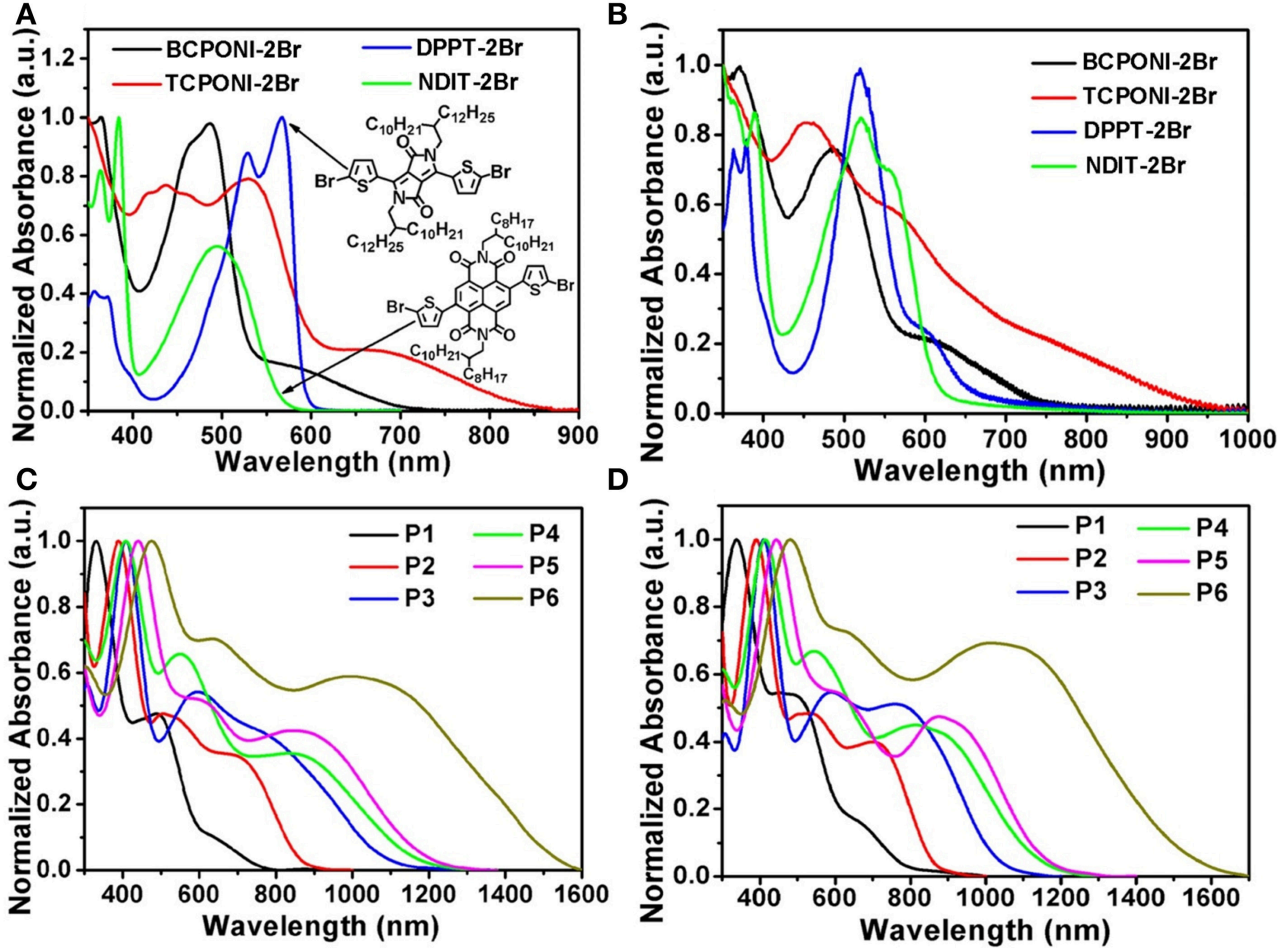
Absorption spectra of the monomers (BCPONI-2Br and TCPONI-2Br), their analogs (DPPT-2Br and NDIT-2Br), and as-synthesized polymers that measured in chloroform solution **(A,C)** and in thin film **(B,D)**.

UV-vis-NIR absorption spectra of the CPONI-containing monomers and their D-A polymers were recorded in chloroform (ca. 10^−5^ M) and in spin-coated thin films (Table 2 and Table S1). As seen from Figure 5A, the absorption spectrum of the BCPONI-2Br solution covers the whole UV-vis band, while the one of TCPONI-2Br is further extended to the NIR band, as far as 850 nm. The maximum absorption peak of the TCPONI-2Br solution is 532 nm, which exhibits a 46 nm red-shift compared with BCPONI-2Br (Figures 5A and Table S1). This bathochromic shift can be ascribed to the enhanced coplanarity and stronger D-A intramolecular interaction between rich-electron thipohene and CPONI moieties. Interestingly, the overwhelmingly electron-deficient feature of the central CPONI acceptor unit endows BCPONI-2Br and TCPONI-2Br with remarkably extended absorption onsets when compared with the well-known acceptor building blocks, such as DPPT-2Br (Gao et al., 2015) and NDIT-2Br (Senkovskyy et al., 2011) (Figures 5A,B). In comparison with solution spectra, both BCPONI-2Br and TCPONI-2Br thin films exhibit much broader absorption bands, with the maximum absorption onsets of ca. 750 and 950 nm, respectively. All these results indicate a strong solid-state aggregation or interchain organization, generally associated with high-mobility charge carrier transport (Zhu et al., 2017).

**Table 2:** Photophysical and electrochemical properties of the Polymers.

**Polymer**	$\lambda_{\max}^{sol}$	$\lambda_{onset}^{sol}$	$\lambda_{max}^{film}$	$\lambda_{onset}^{film}$	$\Delta E_{g}^{opt}$ *^a^*	***E*_*HOMO*_**	$E_{onset}^{ox}$*^b^*	***E*_*LUMO*_**	$E_{onset}^{red}$*^b^*	$\Delta E_{g}^{CV}$
	**(nm)**	**(nm)**	**(nm)**	**(nm)**	**(eV)**	**(eV)**	**(V)**	**(eV)**	**(V)**	**(eV)**
P1	330	762	338	798	1.55	−5.78	1.36	−3.79	−0.63	1.99
P2	389	863	390	860	1.44	−5.34	0.92	−3.77	−0.65	1.57
P3	408	1,088	410	1,032	1.20	−5.12	0.70	−3.76	−0.66	1.36
P4	408	1,174	414	1,152	1.07	−5.43	1.01	−3.88	−0.54	1.55
P5	440	1,196	444	1,164	1.06	−5.40	0.98	−3.88	−0.54	1.52
P6	475	1,534	481	1,530	0.81	−4.96	0.54	−3.86	−0.56	1.10

a *Optical bandgaps estimated from the onset of film absorption and calculated from $\Delta E_{\text{g}}^{\text{opt}}\left(\text{eV}\right)=1,240/\lambda_{\text{onset}}^{\text{film}}$*;

b $E_{\text{onset}}^{\text{ox}}$ and $E_{\text{onset}}^{\text{red}}$ *determined from the first onset of oxidation and reduction potentials, respectively*.

Due to the strong D-A intramolecular interactions (Zhu et al., 2017), all polymers achieve ultra-broadband absorption from UV to NIR (Figures 5C,D). Moreover, three typical absorption bands, corresponding to π-π^*^ transition (ca. 350–500 nm) and charge transfer (ca. 500–1,600 nm), were clearly observed in both solution and thin-film spectra. For TCPONI-based polymers, the maximum absorption peaks were 408 nm for P4, 440 nm for P5, and 475 nm for P6 (Table 2). Compared with TCPONI-containing analogs, three BCPONI-based polymers exhibit relatively narrower light-capturing bands and sharply blue-shifted π-π^*^ transition peaks, which could be explained by the strong backbone twisting and weak electron-donating ability of benzene moieties. In thin film, the π-π^*^ transition peaks for all polymers display a slight (ca. 2–6 nm) red-shift, while the maximum absorption edges were blue-shifted by ca. 4–56 nm (Table 2), indicative of a more planar conformation in their solid-state films (Zhu et al., 2017). Accordingly, all these observations reveal that the absorption and $E_{\text{g}}^{\text{opt}}$ of the BCPONI- and TCPONI-containing polymers can be fine-tuned by choosing different donors and π-conjugation linkers. Moreover, the absorption wavelength can be extended easily by increasing the electron-donating ability of the donor units. On the basis of the absorption onsets of polymer films, the $E_{\text{g}}^{\text{opt}}$ values were determined to be 1.55 eV for P1, 1.44 eV for P2, 1.20 eV for P3, 1.07 eV for P4, 1.06 eV for P5, and 0.81 for P6. Notably, such ultralow $E_{\text{g}}^{\text{opt}}\;\approx$ 0.81 observed here suggests that P6 has a highly delocalization of the π-electrons, which can be associated with good backbone coplanarity, large π-conjugation, and strong D-A interaction between donor and CPONI acceptor moieties (Chen et al., 2012; Zhu et al., 2017).

### Electrochemical Properties

**Figure 6:**
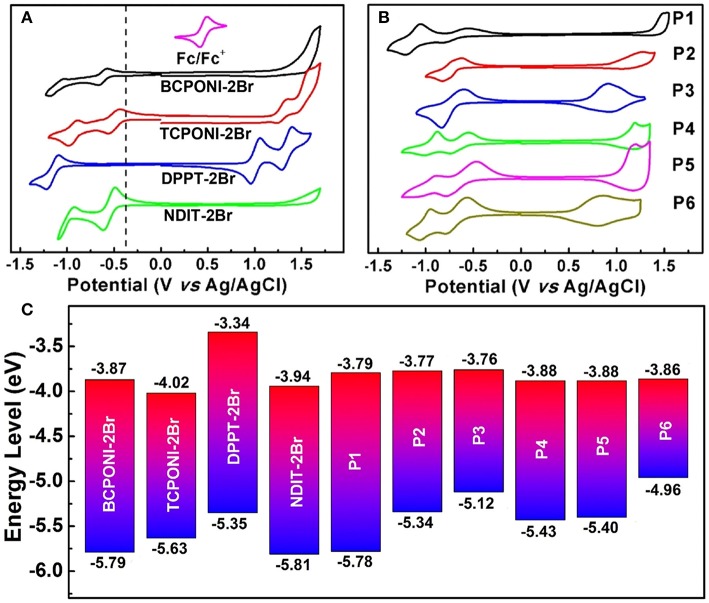
**(A)** CV curves of the monomers (BCPONI-2Br and TCPONI-2Br), their analogs (DPPT-2Br and NDIT-2Br). **(B)** CV curves of the polymers. **(C)** Comparative diagram for the HOMO and LUMO energy levels.

To evaluate the electrochemical properties of the CPONI-containing monomers and their D-A polymers, CV measurements were performed in both dichloromethane solution and thin film. Detailed CV data are provided in Table 2, Table S1, and Figure 6. The *E*_HOMO_ and *E*_LUMO_ levels are calculated from the onset oxidation ($E_{onset}^{ox}$) and reduction ($E_{onset}^{red}$) potentials using the following equations: *E*_HOMO_ = –($E_{onset}^{ox}$ + 4.42) (eV) and *E*_LUMO_ = –($E_{onset}^{red}$ + 4.42) (eV), which is calibrated by ferrocene/ferrocenium (Fc/Fc^+^) couple (0.38 V vs. Ag/AgCl) (Chen et al., 2012; Zhu et al., 2017). During positive and negative scans, reversible oxidation, and reduction processes were observed for TCPONI-2Br, while only reduction processes show a reversible feature for BCPONI-2Br (Figure 6A). The calculated LUMO and HOMO energy levels of BCPONI-2Br are −3.87 and −5.79 eV, respectively. In comparison with benzene-flanked BCPONI-2Br, thiophene-flanked TCPONI-2Br exhibits a reduced LUMO energy level (−4.02 eV) but an elevated HOMO energy level (−5.63 eV), owing to enhanced molecular coplanarity as well as improved electron-donating ability of thiophene units (Cui and Wudl, 2013). Surprisingly, much deeper LUMO energy levels for both monomers are observed than that of the previously reported acceptor unit DPPT-2Br (LUMO = −3.34 eV) (Gao et al., 2015), and are even comparable to the classical *n*-type building block NDIT-2Br (LUMO = −3.94 eV) (Senkovskyy et al., 2011). These results suggest that BCPONI-2Br and TCPONI-2Br are very strong acceptor units, which exhibits great potential in construction of various organic/polymeric electron-transporting materials.

All the polymers P1–P6 exhibit strong and reversible oxidation and reduction processes (Figure 6B). The LUMO energy levels of P1–P6, estimated from $E_{onset}^{red}$, are −3.79 eV for P1, −3.77 eV for P2, −3.76 eV for P3, −3.88 eV for P4, −3.88 eV for P5, and −3.86 eV for P6. As to two type of polymers, their LUMO energy levels show a negligible change with the enhancement of the electron-donating capability of the donor units, while they can be directly influenced by the π-conjugation linkers of both BCPONI-2Br and TCPONI-2Br monomers; therefore, the thiophene-flanked P4–P6 show slightly reduced LUMO energy levels relative to the benzene-flanked analogs (P1–P3). It was found that the HOMO energy levels and band gaps of the polymers can be readily tuned by the selection of different donors. With increasing donor strength, the HOMO energy levels of the polymers will be upshifted, which caused a reduced band gap. Additionally, the electrochemical band gaps $\left(E_{\text{g}}^{\text{cv}}\right)$ determined here are ca. 0.13–0.48 eV higher than those of their $E_{\text{g}}^{\text{opt}}$. Such a small difference between $E_{\text{g}}^{\text{cv}}$ and $E_{\text{g}}^{\text{opt}}$ has been reported in many studies (Cui and Wudl, 2013 and Chen et al., 2016) and can be explained by the exciton binding energy of the π-conjugated polymers (Sariciftci, 1997).

### OFET Performance and Film Organization

**Figure 7:**
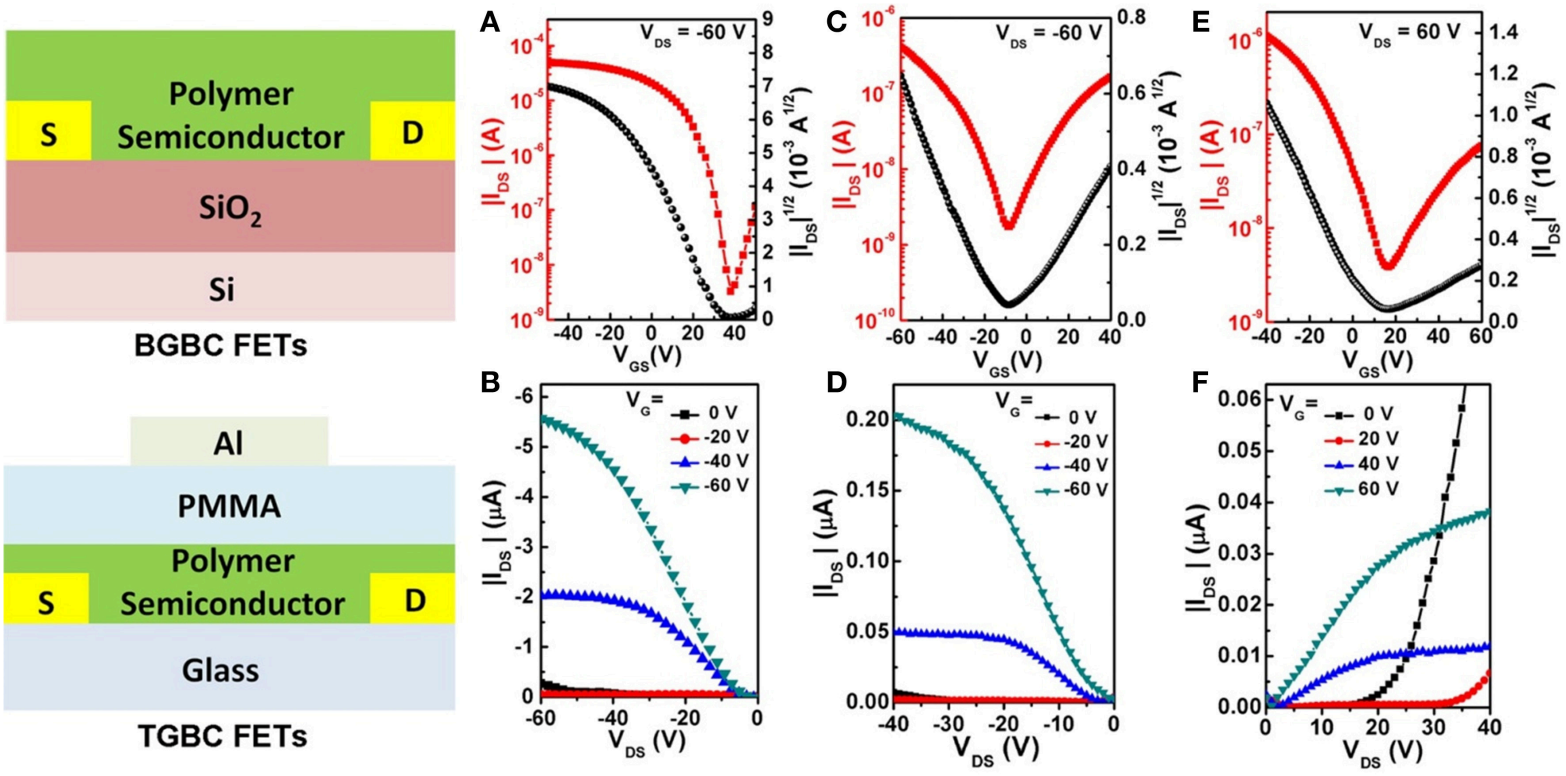
**(A)** Transfer and **(B)** output curves of the BGBC OFET devices. **(C,E)** Transfer and **(D,F)** output curves of the TGBC OFET devices.

To demonstrate the application potential of the CPONI-based polymers in OFETs, P5 was chosen as an example to fabricate polymer FET devices due to its good backbone coplanarity, proper HOMO/LUMO energy levels, good solubility, and high molecular weight. For the optimization of charge carrier transport performance, both bottom-gate/bottom-contact (BG/BC) and top-gate/bottom-contact (TG/BC) device configurations were used to fabricate polymeric FETs. The detailed device fabrication procedures can be found in the supporting information. Under ambient conditions, P5-based BG/BC OFETs exhibited a typical *p*-type transport characteristic with a moderately high hole mobility of 0.02 cm^2^ V^−1^ s^−1^ and current on/off ratio >10^4^, while only weak electron transport can be observed in both transfer and output curves (Figures 7A,B). Considering that the LUMO value (−3.88 eV) of P5 is far from the requirement of thermodynamic stabile electron transport, electrons can be readily captured by H_2_O/O_2_ in air (Zhan et al., 2011 and Chen et al., 2013). Therefore, only strong hole transport were observed in the P5-based BG/BC OFETs. Due to an effective encapsulation effect of the dielectric layer in TG/BC OFET devices, P5 exhibited an obvious ambipolar transport behavior with both *p*- and *n*-type operation modes for negative and positive gate voltages, respectively. The saturation mobilities were determined to be 4.11 × 10^−3^ cm^2^ V^−1^ s^−1^ for holes and 5.15 × 10^−4^ cm^2^ V^−1^ s^−1^ for electrons (Figures 7C–F). The observed hole and electron mobilities are sufficient for charge carrier transport in potential OPV devices, especially in all-polymer OPVs (Cui and Wudl, 2013). The findings presented above suggest that BCPONI-2Br and TCPONI-2Br are promising building blocks for the construction of polymer electron-transporting materials with attractive electronic properties. To further characterize film organization and surface morphology, AFM and GIXRD measurements were conducted. As seen from Figure S10A, the π-π stacking reflection (010) are clearly observed in the *q*_z_ direction, indicative of a primarily face-on model packing for the P5 film. This type of stacking model is consistent with the classical NDI-based polymers such as N2200 (Yan et al., 2009). The calculated lamellar stacking distance and π-π stacking distance are around 29.11 and 3.79 Å, which were determined from the (100) and (010) peaks, respectively. Furthermore, P5 film shows a smooth surface microscopy and a very small root-mean-square surface roughness of 0.82 nm (Figure S10B), which is helpful for good interface contact between polymer film and dielectric layer (Zhu et al., 2017).

## Conclusions

We have designed and synthesized two novel aromatic imides, diarylcyclopentadienone-fused naphthalimides (BCPONI-2Br and TCPONI-2Br), in which a five-membered cyclopentadienone unit is fused at the lateral position of the naphthalimide skeleton. Compared with both well-known DPPT-2Br and NDIT-2Br building blocks, BCPONI-2Br and TCPONI-2Br exhibit extended absorption bands, narrower band gaps, and even lower LUMO levels, as low as −4.02 eV. Such deep-lying LUMO values enable them to function as the strong acceptors. Furthermore, Stille and Suzuki polycondensation between both novel acceptors and different donors (carbazole, benzodithiophene, and dithienopyrrole) was performed to afford six novel D–A polymers (P1–P6). It was found that optical and electrochemical properties of the polymers are fine-tuned by the variations of donors and π-conjugation linkers. Compared with the BCPONI-containing analogs (P1–P3), the TCPONI-containing P4–P6 exhibit extended absorption bands and deeper LUMO energy levels due to more electron-rich thiophene and more planar π-conjugation skeleton. With an increasing electron-donating ability of the donor units, an extended NIR absorption and an upshifted HOMO levels were observed clearly, while LUMO levels were almost unaffected. The estimated LUMO levels were as low as −3.88 eV, indicating very strong electron affinities for these polymers. Preliminary OFETs results show that P5 exhibits a moderately high hole mobility of 0.02 cm^2^ V^−1^ s^−1^ in BGBC OFETs and a typical ambipolar transport behavior in TGBC OFETs. All these observed results suggest that BCPONI-2Br and TCPONI-2Br units are very strong and interesting acceptor building blocks for the creation of various low bandgap π-conjugated materials, especially for electron-transporting polymers. These polymers could be functioned as the electron acceptor materials in all-polymer solar cells or other optoelectronic devices.

## Data Availability

All datasets generated for this study are included in the manuscript and/or the Supplementary Files.

## Author Contributions

HC and XL designed, synthesized, and characterized polymeric semiconductors. JG, LY, YH, and YZ measured OFET devices. LZ conducted the CV experiments. ZM performed DFT calculations. All authors were responsible for discussing the results. HC designed experiments and wrote the manuscript.

### Conflict of Interest Statement

The authors declare that the research was conducted in the absence of any commercial or financial relationships that could be construed as a potential conflict of interest.
