# Supplementary material for: Low Bandgap Donor-Acceptor π-Conjugated Polymers From Diarylcyclopentadienone-Fused Naphthalimides
Source: Front Chem. 2019 May 29;7:362. doi: 10.3389/fchem.2019.00362 (PMC6549120; doi:10.3389/fchem.2019.00362)
Supplement: Supplementary file 1 [file Data_Sheet_1.docx]

Supplementary Material

**Low Bandgap Donor-Acceptor π-Conjugated Polymers**

**From Diarylcyclopentadienone-Fused Naphthalimides**

**Xiaolin Li^1^, Jing Guo^2^, Longfei Yang^3^, Minghao Chao^3^, Liping Zheng^1^, Zhongyun Ma^1^, Yuanyuan Hu^2,*^, Yan Zhao^3,*^, Huajie Chen^1,*^, and Yunqi Liu^3^**

^1^*Key Laboratory for Green Organic Synthesis and Application of Hunan Province, and Key Laboratory of Environmentally Friendly Chemistry and Applications of Ministry of Education, College of Chemistry, Xiangtan University, Xiangtan 411105, China*.

^2^*Key Laboratory for Micro/Nano Optoelectronic Devices of Ministry of Education & Hunan Provincial Key Laboratory of Low-Dimensional Structural Physics and Devices, School of Physics and Electronics, Hunan University, Changsha 410082, China.*

^3^*Institute of Molecular Materials and Devices, Department of Materials Science, Fudan University, Shanghai, 200433, China*

**1. Device Fabrication and Mobility Measurements**

Bottom-gate/bottom-contact (BG/BC) field-effect transistor (FET) devices were fabricated on Si/SiO_2_ substrates. Photolithography was used to prepare source/drain electrodes (titanium/gold: 5 nm/30 nm) with the channel length (*L*) and channel width (*W*) of 5 and 1400 *μ*m, respectively. The substrates were subjected to ultrasonication cleaning in acetone, deionized water, and ethanol in sequence. After that, the substrates were modified by trichloro(octadecyl)silane (OTS) to reduce interface traps. The semiconducting layer was deposited on the substrates by spin-coating a polymer solution (6 mg/mL in chlorobenzene), and then annealed at 160 °C for 30 mins.

For top-gate/bottom-contact (TG/BC) FET devices, the bottom-contact electrodes (Cr/Au: 2 nm/ 30 nm) were prepared by photolithography, which provides the *L* and *W* of 20 and 1000 *μ*m, respectively. Then semiconducting layer was spin-coated and annealed as mentioned above, followed by the deposition of PMMA (90 mg/mL in *n*-Butyl Acetate, 2000 rpm) as dielectrics. The thickness of PMMA is about 770 nm, as determined by AFM. After that, 30 nm aluminum was deposited on top of the dielectric to serve as the gate electrodes. The charge carrier transport performance of the devices was characterized by a Keithley 4200 SCS semiconductor parameter analyzer under ambient conditions.

The field-effect mobility in saturation (*μ*) is calculated from the following equation:

*I*_DS_ = (*W*/2*L*) *C*_i_*μ*(*V*_GS_–*V*_TS_)^2^

where *W*/*L* is the channel width/length, *C*_i_ is the gate dielectric layer capacitance per unit area, and *V*_GS_ and *V*_TS_ are the gate voltage and threshold voltage, respectively.

**2. GPC analysis**

**
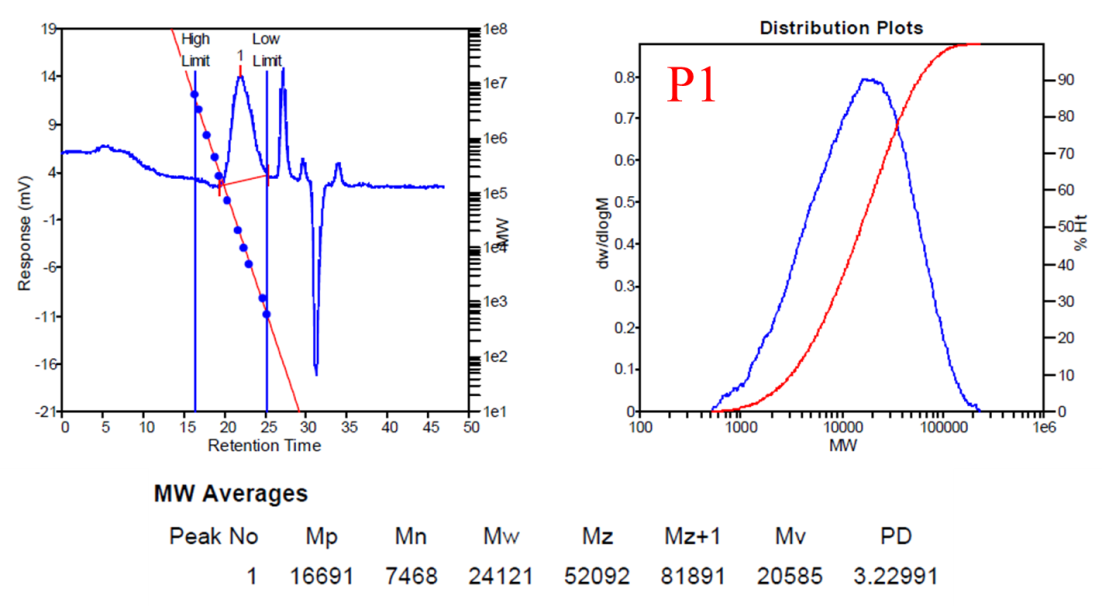
**

**Figure S1.** GPC curves of **P1**.

**
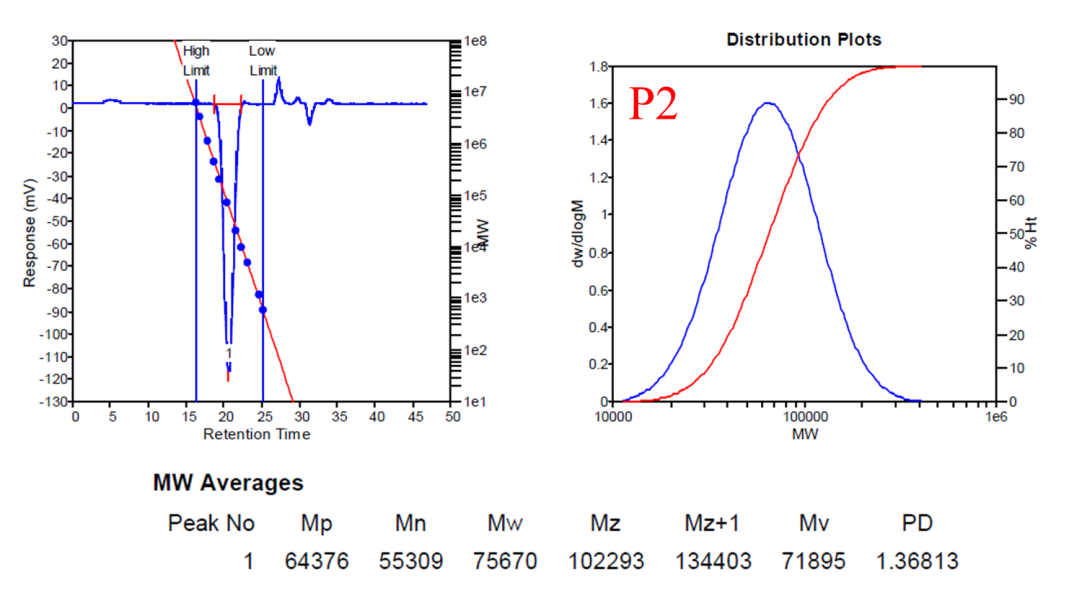
**

**Figure S2.** GPC curves of **P2**.

**
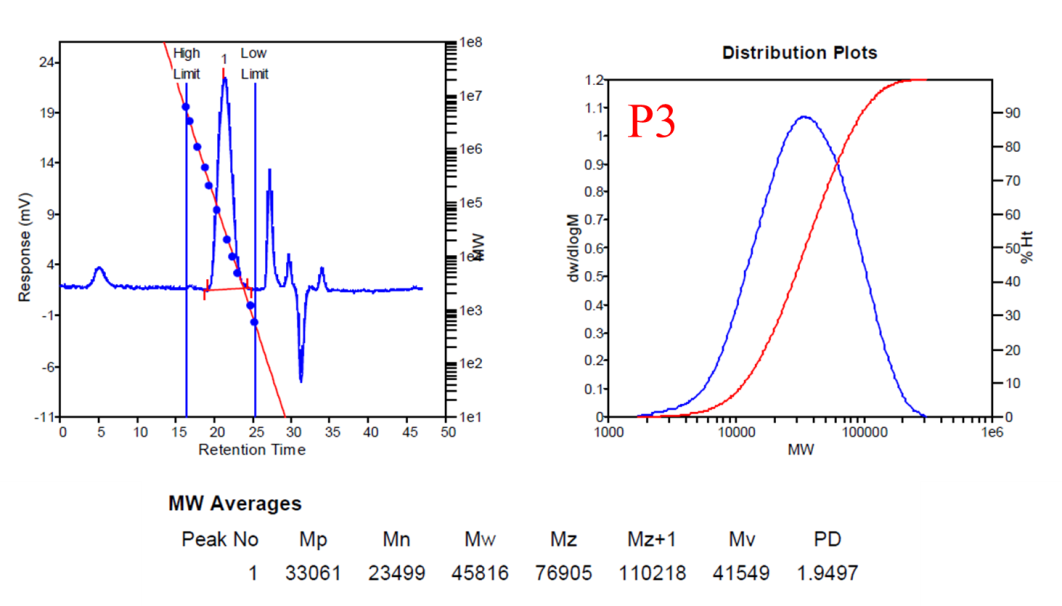
**

**Figure S3.** GPC curves of **P3.**

**
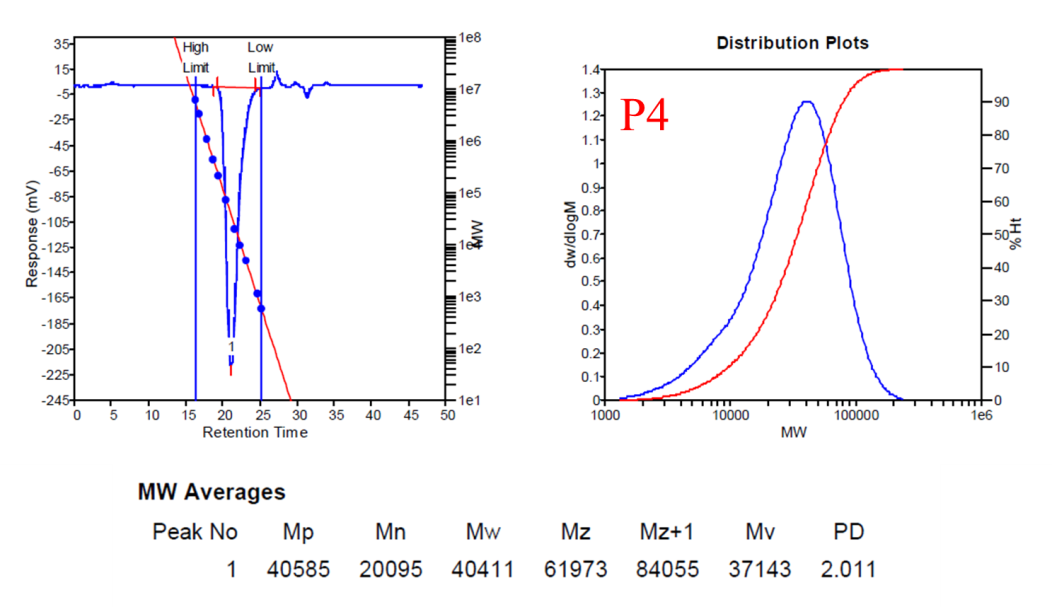
**

**Figure S4.** GPC curves of **P4.**

**
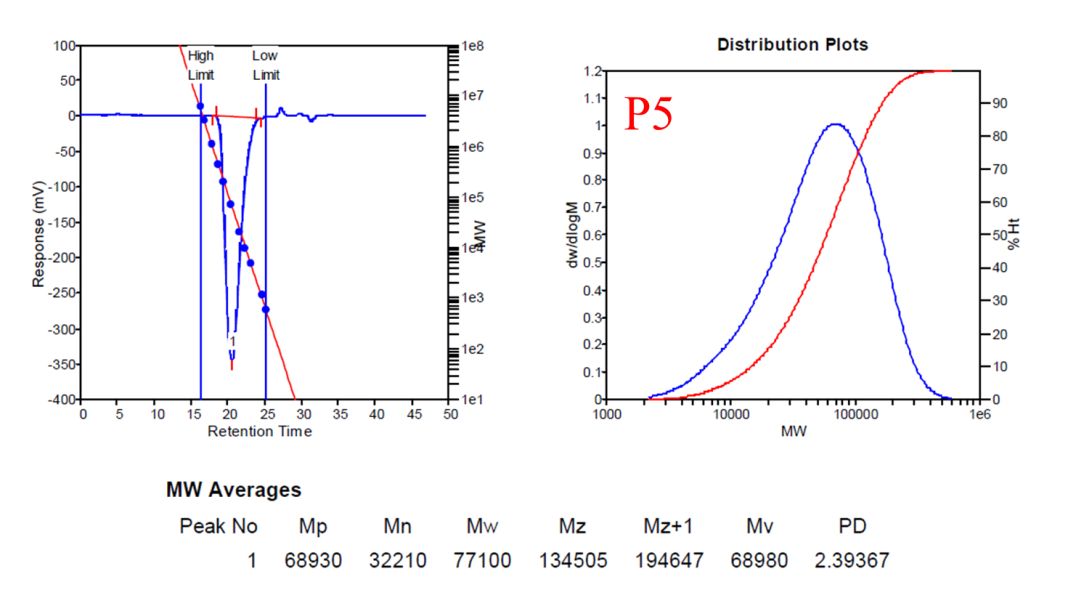
 Figure S5.** GPC curves of **P5.
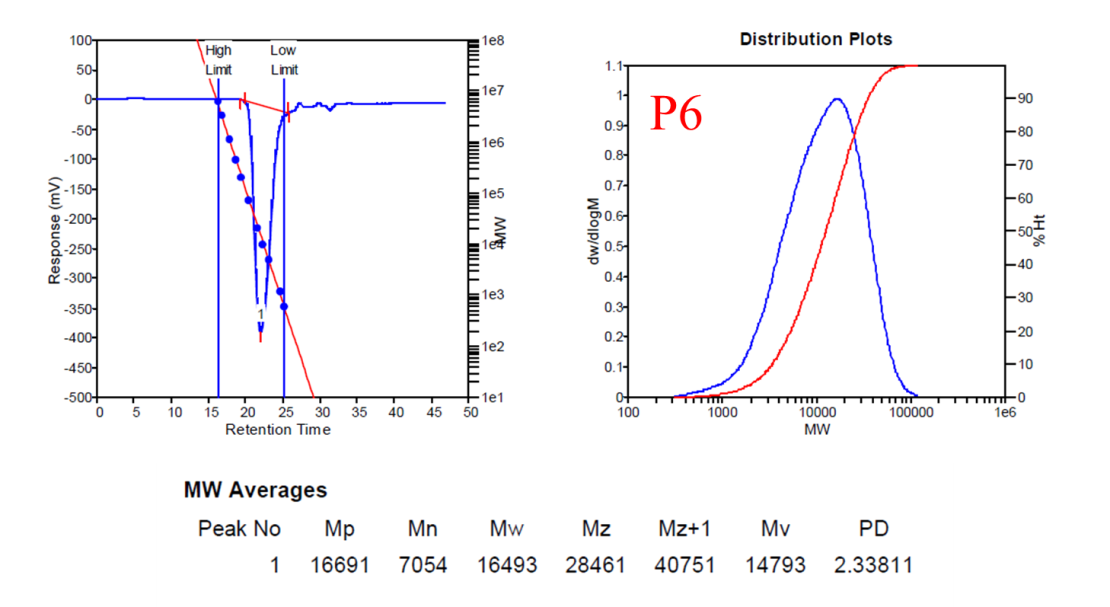
**

**Figure S6.** GPC curves of **P6**.

#

# 3. Thermal properties (DSC)

**Figure S7.** DSC curves of the polymers P1-P6.

#

# 4. FT-IR spectra analysis

**
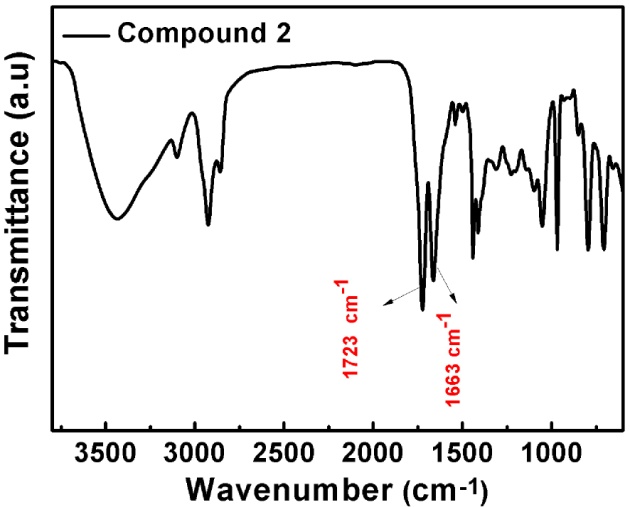
**
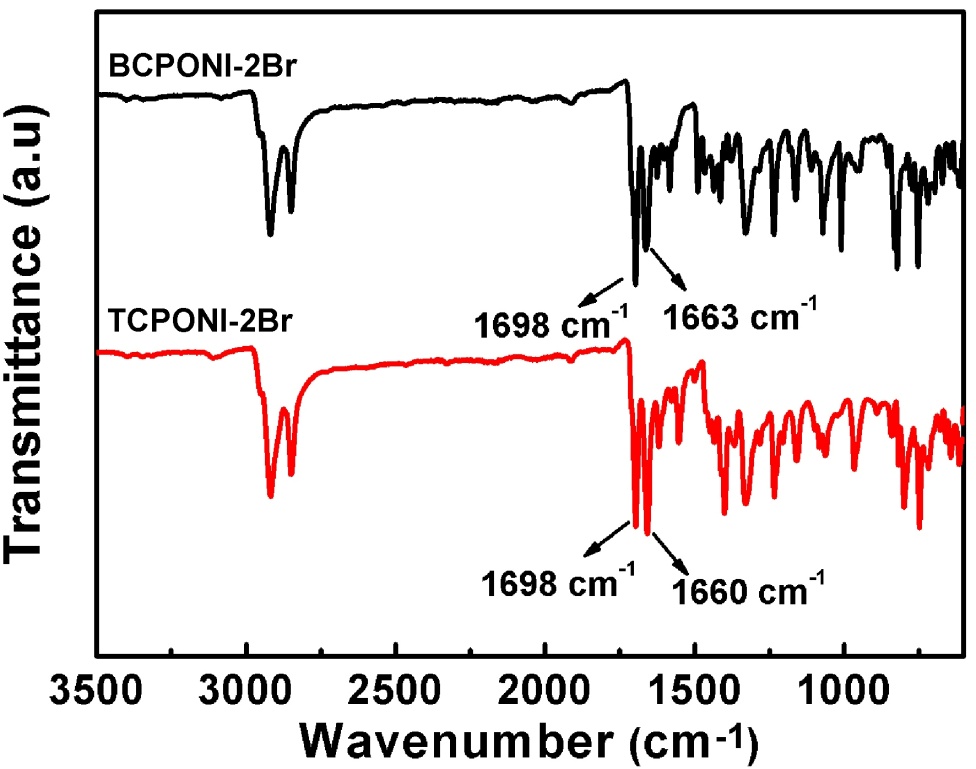


**Figure S8.** FT-IR spectra of the compounds 2, BCPONI-2Br, and TCPONI-2Br.


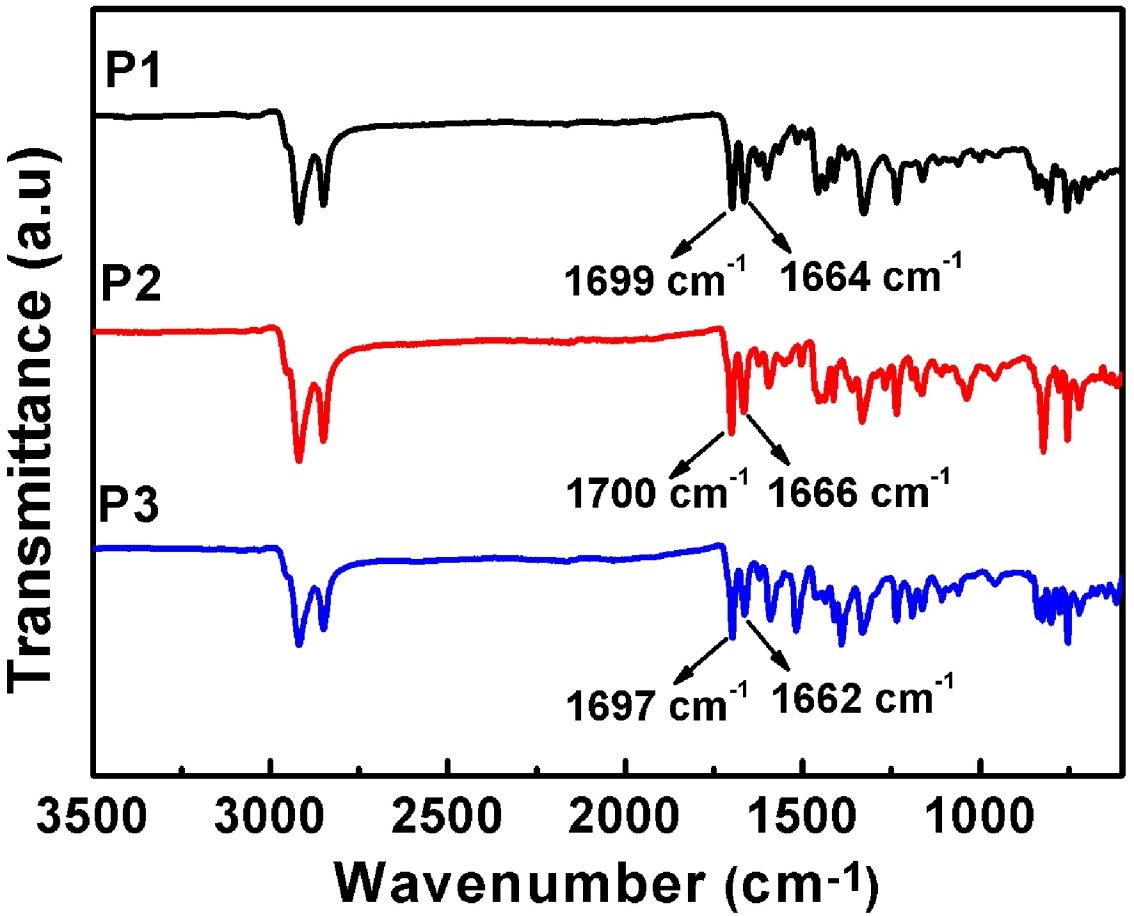

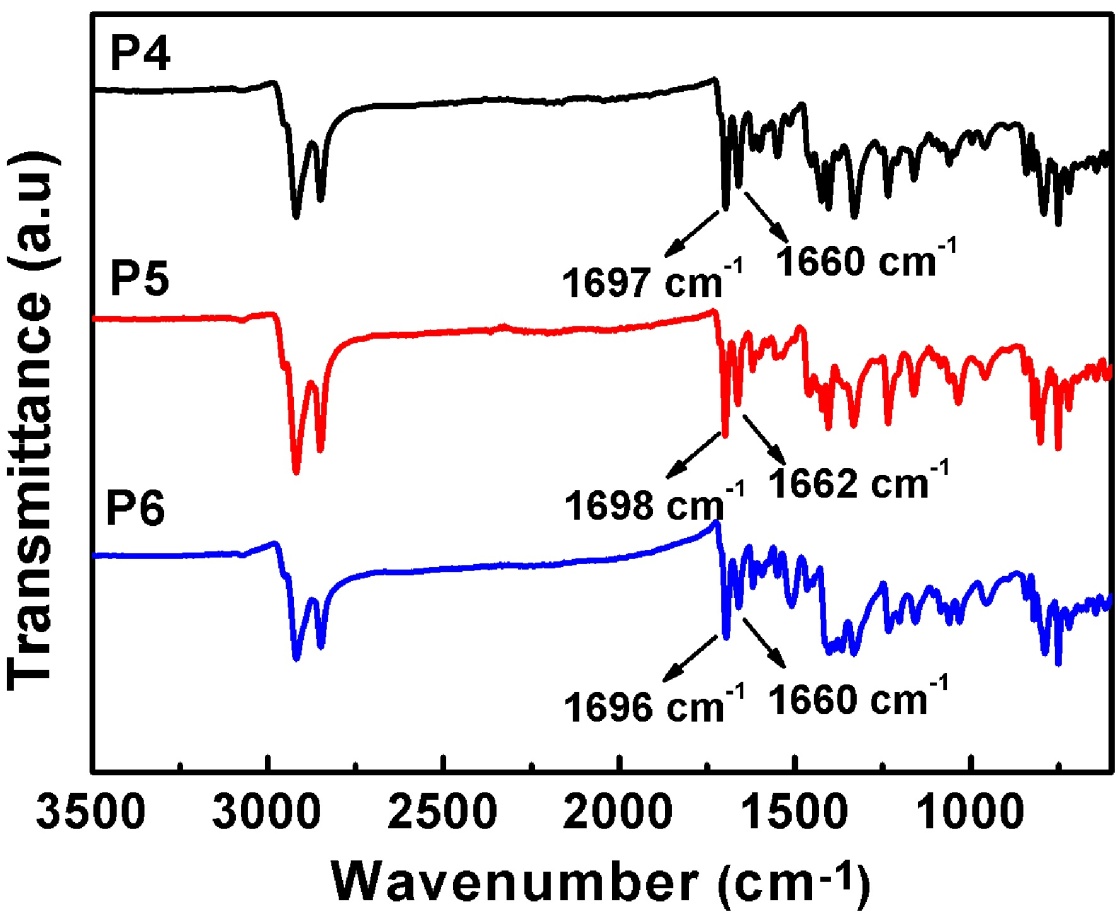


**Figure S9.** FT-IR spectra of the polymers **P1-P6**.

# 5. Table S1. Photophysical and electrochemical properties of the CPONI-containing monomers and their analogues.

| **Monomer** | $\boldsymbol{\lambda}_{\mathbf{max}}^{\mathbf{sol}}$  **(nm)** | $\boldsymbol{\lambda}_{\mathbf{onset}}^{\mathbf{sol}}$  **(nm)** | $\boldsymbol{\lambda}_{\mathbf{max}}^{\mathbf{film}}$  **(nm)** | $\boldsymbol{\lambda}_{\mathbf{onset}}^{\mathbf{film}}$  **(nm)** | ${\boldsymbol{\Delta}\boldsymbol{E}}_{\mathbf{g}}^{\mathbf{opt}}$  **(eV)** | $\boldsymbol{E}_{\mathbf{HOMO}}$  **(eV)** | $\boldsymbol{E}_{\mathbf{onset}}^{\mathbf{ox}}$  **(V)** | $\boldsymbol{E}_{\mathbf{LUMO}}$  **(eV)** | $\boldsymbol{E}_{\mathbf{onset}}^{\mathbf{re}}$  **(V)** | ${\boldsymbol{\Delta}\boldsymbol{E}}_{\mathbf{g}}^{\mathbf{cv}}$  **(eV)** |
| --- | --- | --- | --- | --- | --- | --- | --- | --- | --- | --- |
| BCPONI-2Br | 486 | 702 | 484 | 760 | 1.63 | -5.79 | 1.37 | -3.87 | -0.55 | 1.92 |
| TCPONI-2Br | 530 | 842 | 451 | 949 | 1.31 | -5.63 | 1.21 | -4.02 | -0.40 | 1.61 |
| DPPT-2Br | 567 | 600 | 520 | 666 | 1.86 | -5.35 | 0.93 | -3.34 | -1.08 | 2.01 |
| NDIT-2Br | 494 | 579 | 521 | 634 | 1.96 | -5.81 | 1.39 | -3.94 | -0.48 | 1.87 |

# 6. GIXRD and AFM data.


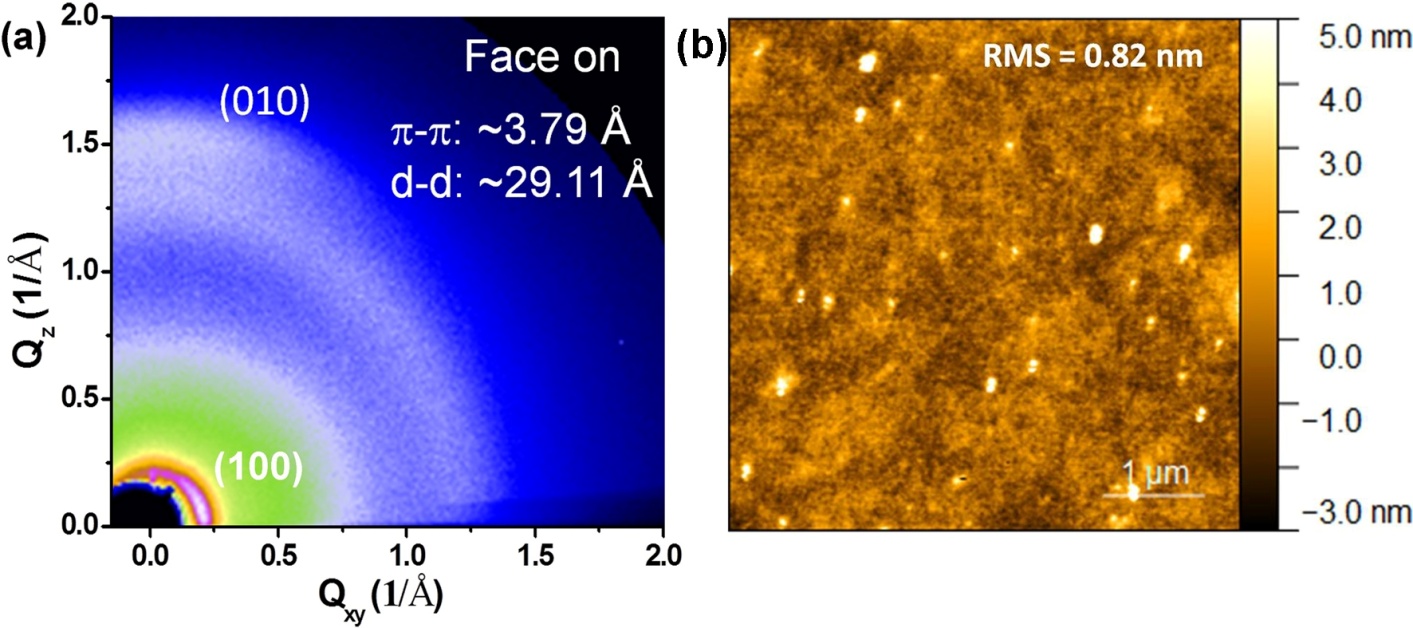
 **Figure S10.** GIXRD data (a) and AFM high image (b) of the P5 films deposited on the OTS-modified SiO_2_/Si substrates. All the P5 films were annealed at 160 °C for 30 min.

# 7. ^1^H NMR and ^13^C NMR spectra.


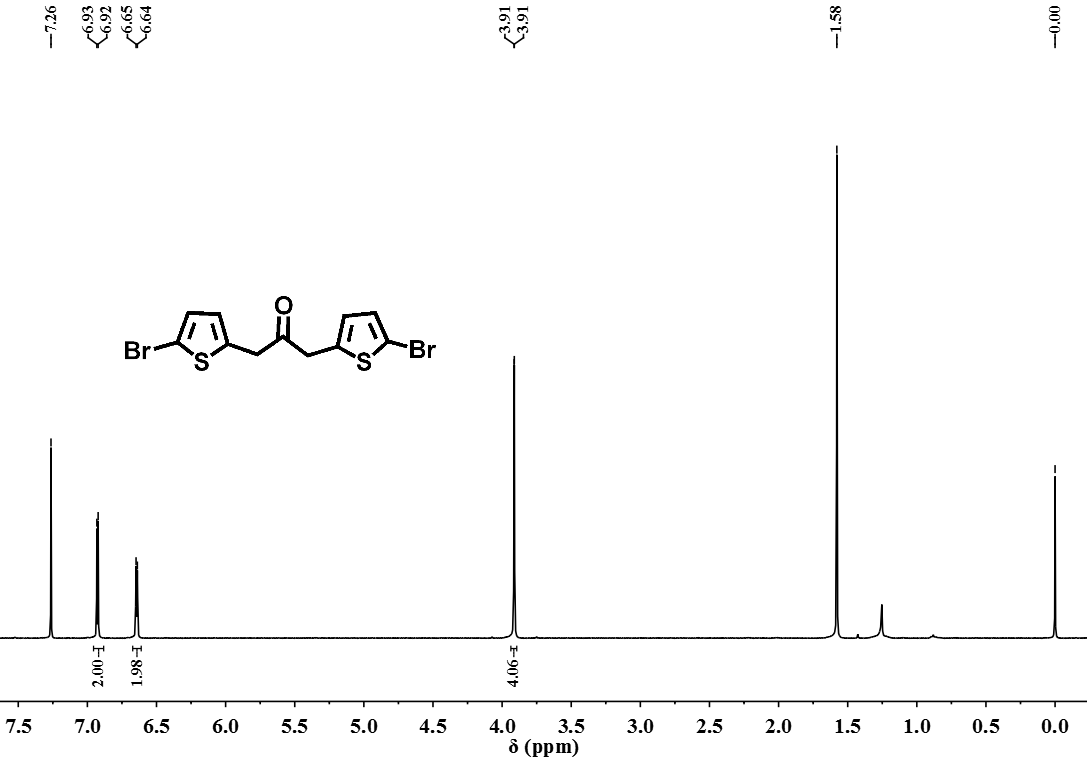


**Figure S11.** 400 MHz ^1^H NMR spectrum of **2** measured in CDCl_3_ at 298K.

**Figure S12.** 100 MHz ^13^C NMR spectrum of **2** measured in CDCl_3_ at 298K.


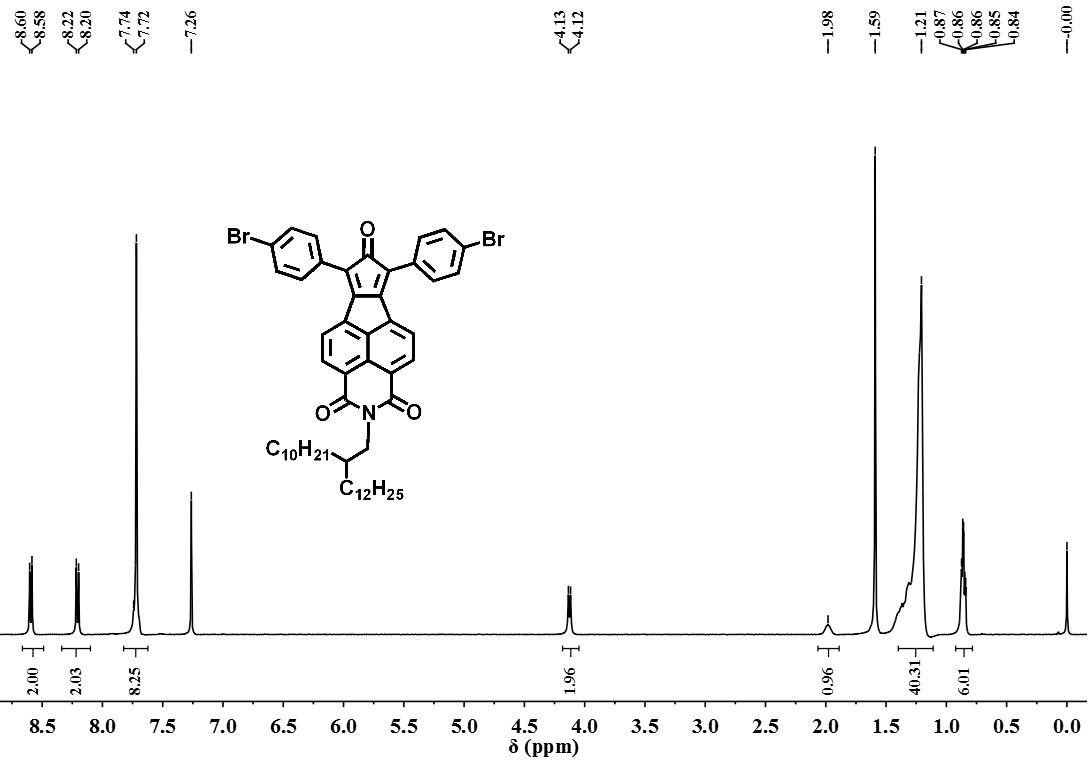


**Figure S13.** 400 MHz ^1^H NMR spectrum of **BNDIO**-**2Br** measured in CDCl_3_ at 298K.


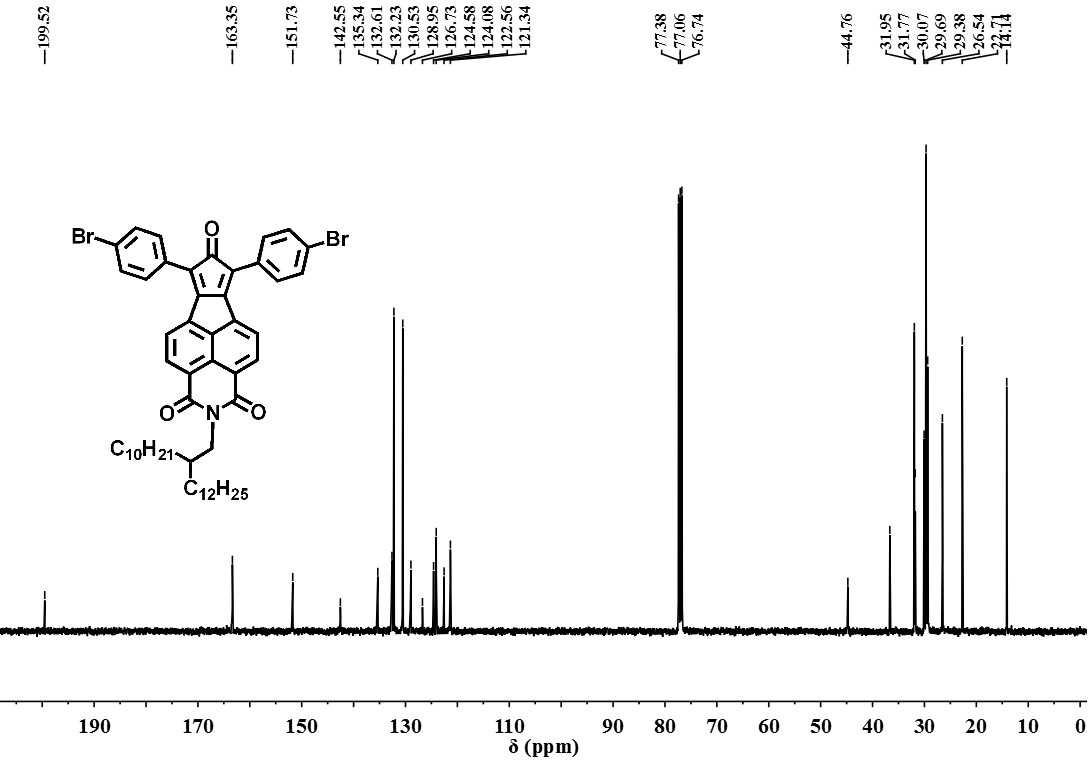


**Figure S14.** 100 MHz ^13^C NMR spectrum of **BNDIO**-**2Br** measured in CDCl_3_ at 298K.


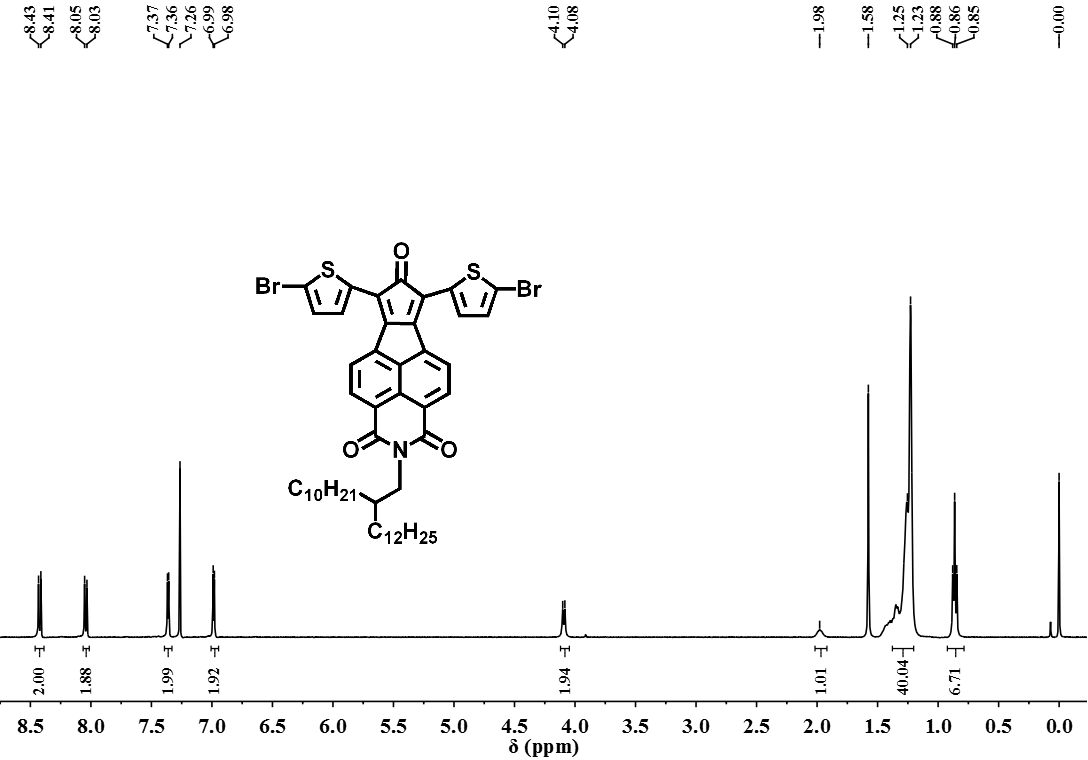


**Figure S15.** 400 MHz ^1^H NMR spectrum of **TNDIO**-**2Br** measured in CDCl_3_ at 298K.


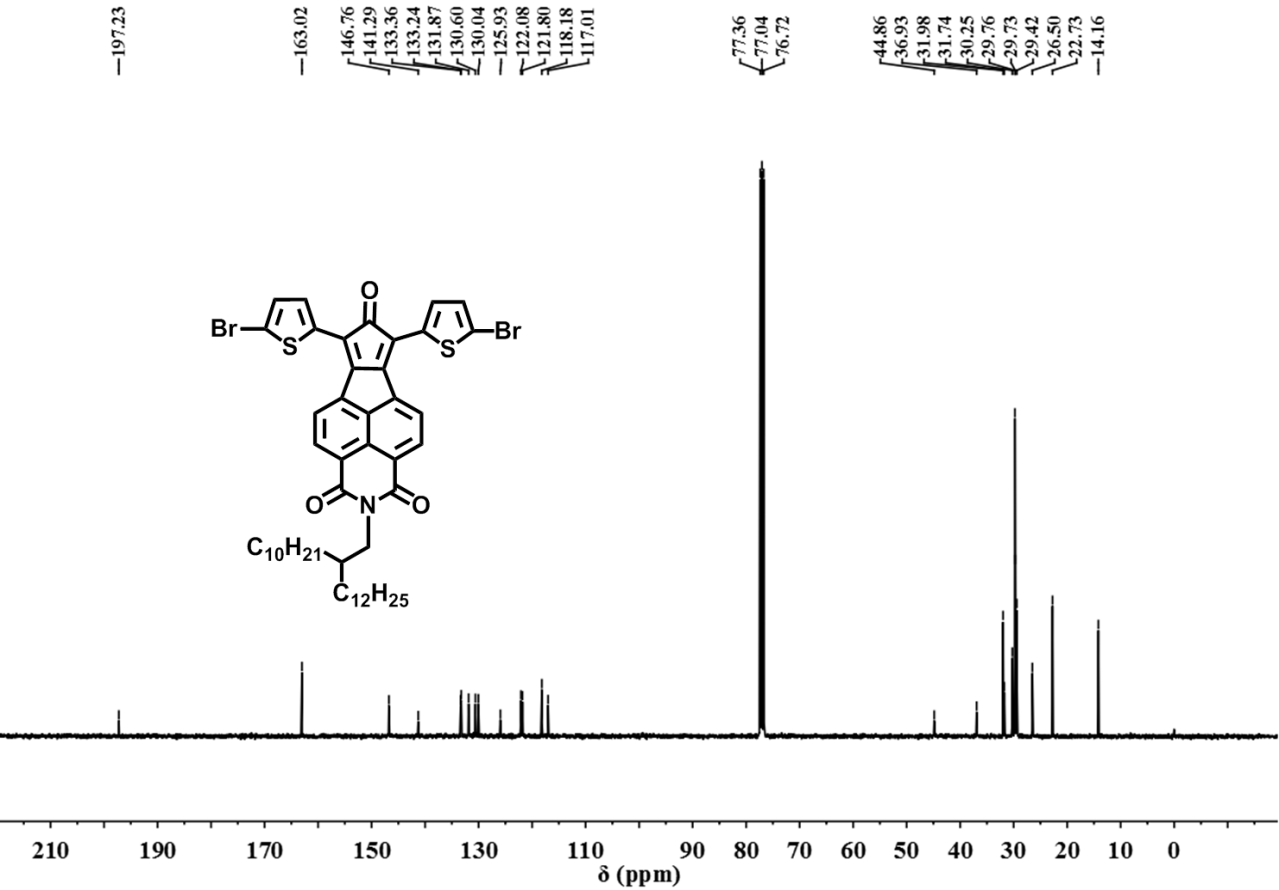


**Figure S16.** 100 MHz ^13^C NMR spectrum of **TNDIO**-**2Br** measured in CDCl_3_ at 298K.


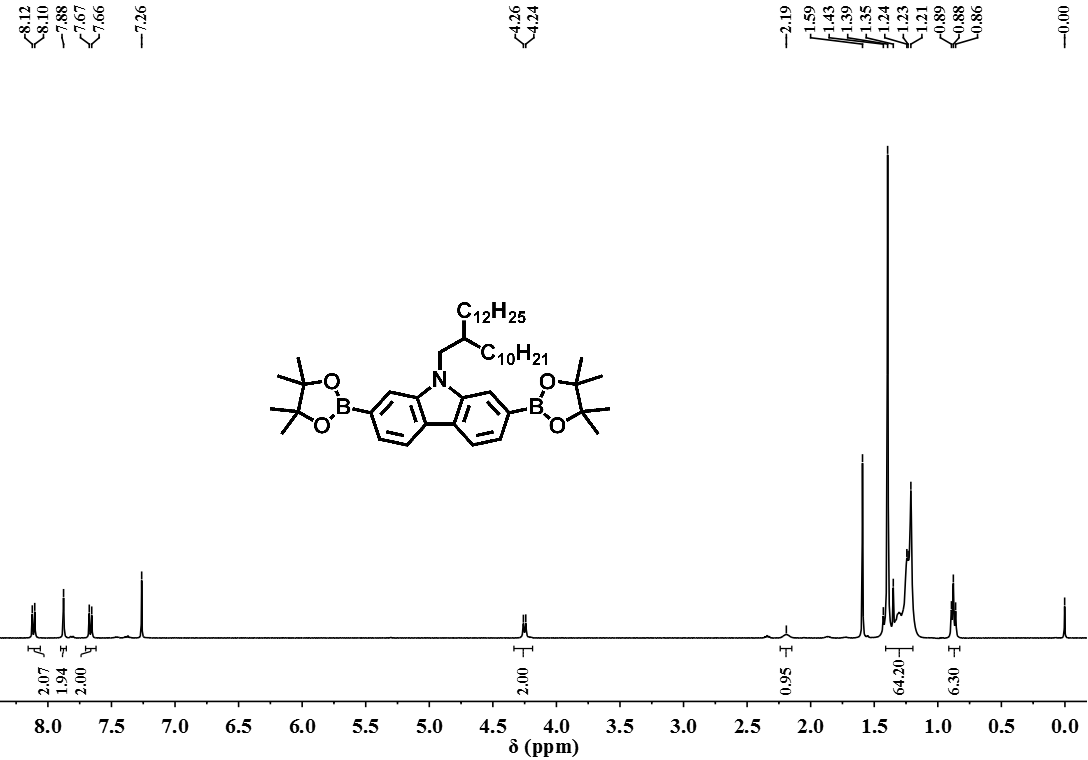


**Figure S17.** 400 MHz ^1^H NMR spectrum of *N*-(2-decyltetradecyl)-2,7-bis-(4,4,5,5-tetramethyl-1,3,2-dioxaborolane-2-yl)carbazole measured in CDCl_3_ at 298K.


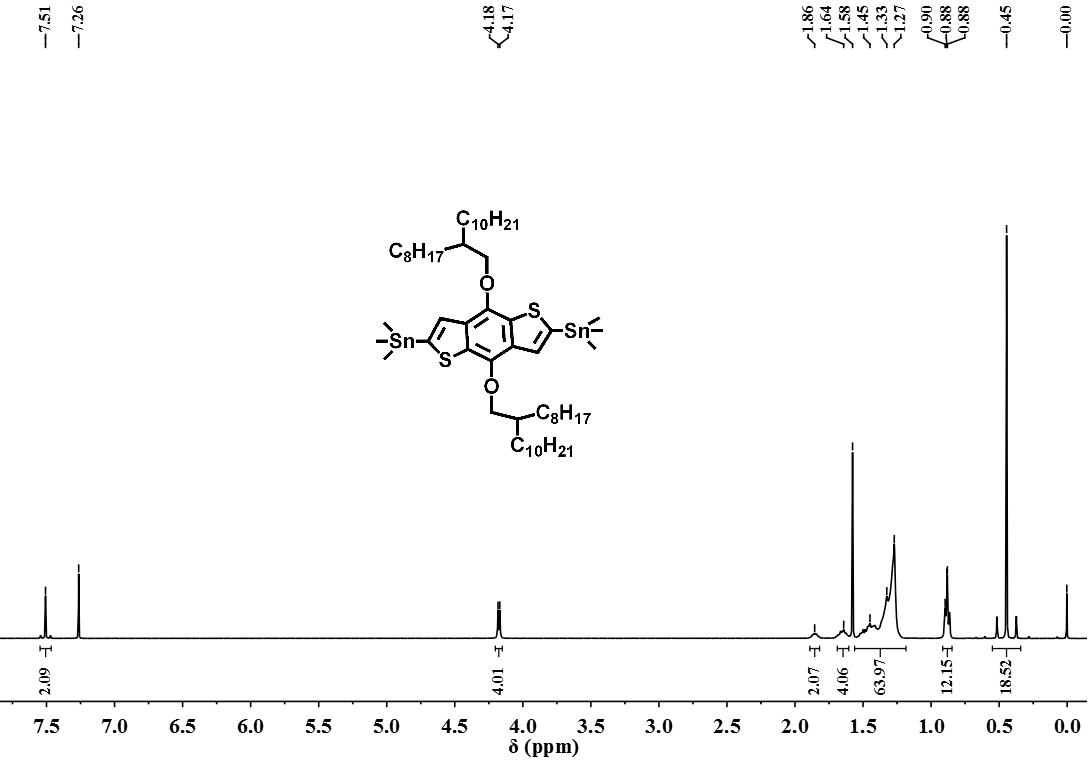


**Figure S18.** 400 MHz ^1^H NMR spectrum of 2,6-bis(trimethyltin)-4,8-di(2-hexyl)decyloxybenzo[1,2-*b*;3,4-*b*’]dithiophene measured in CDCl_3_ at 298K.


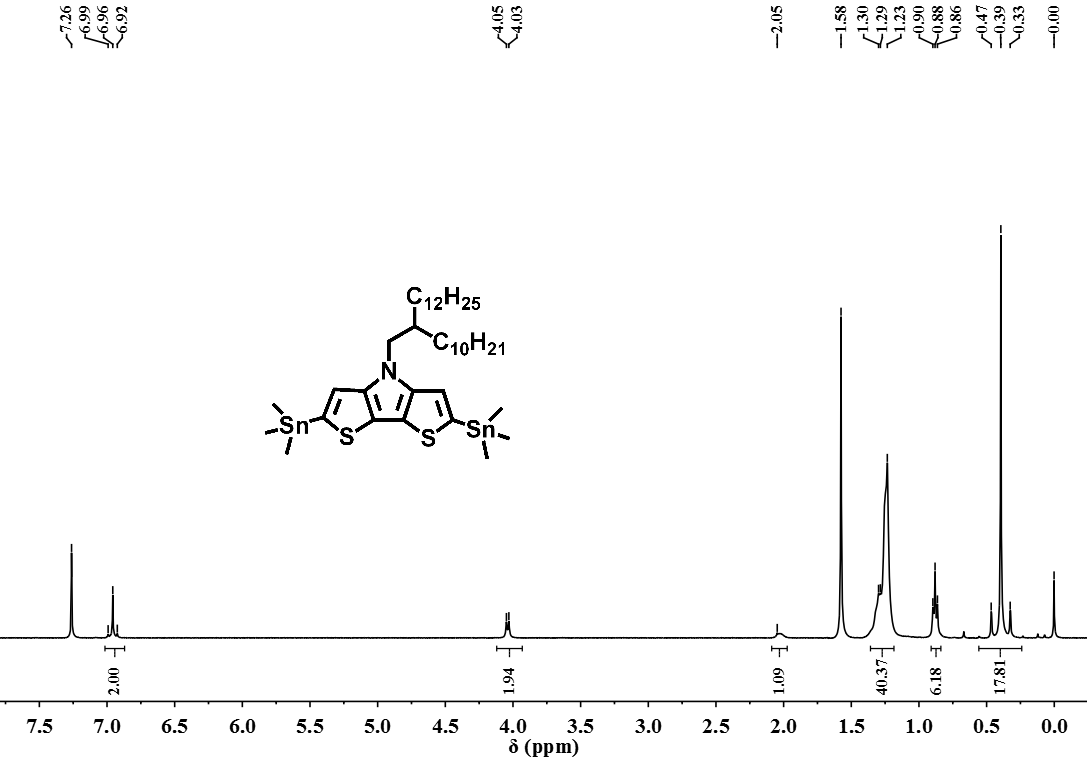


**Figure S19.** 400 MHz ^1^H NMR spectrum of 2,6-bis(trimethylstannyl)-*N*-(2-decyltetradecyl)-dithieno[3,2-*b*:2’,3’-*d*]pyrrole measured in CDCl_3_ at 298K.

**
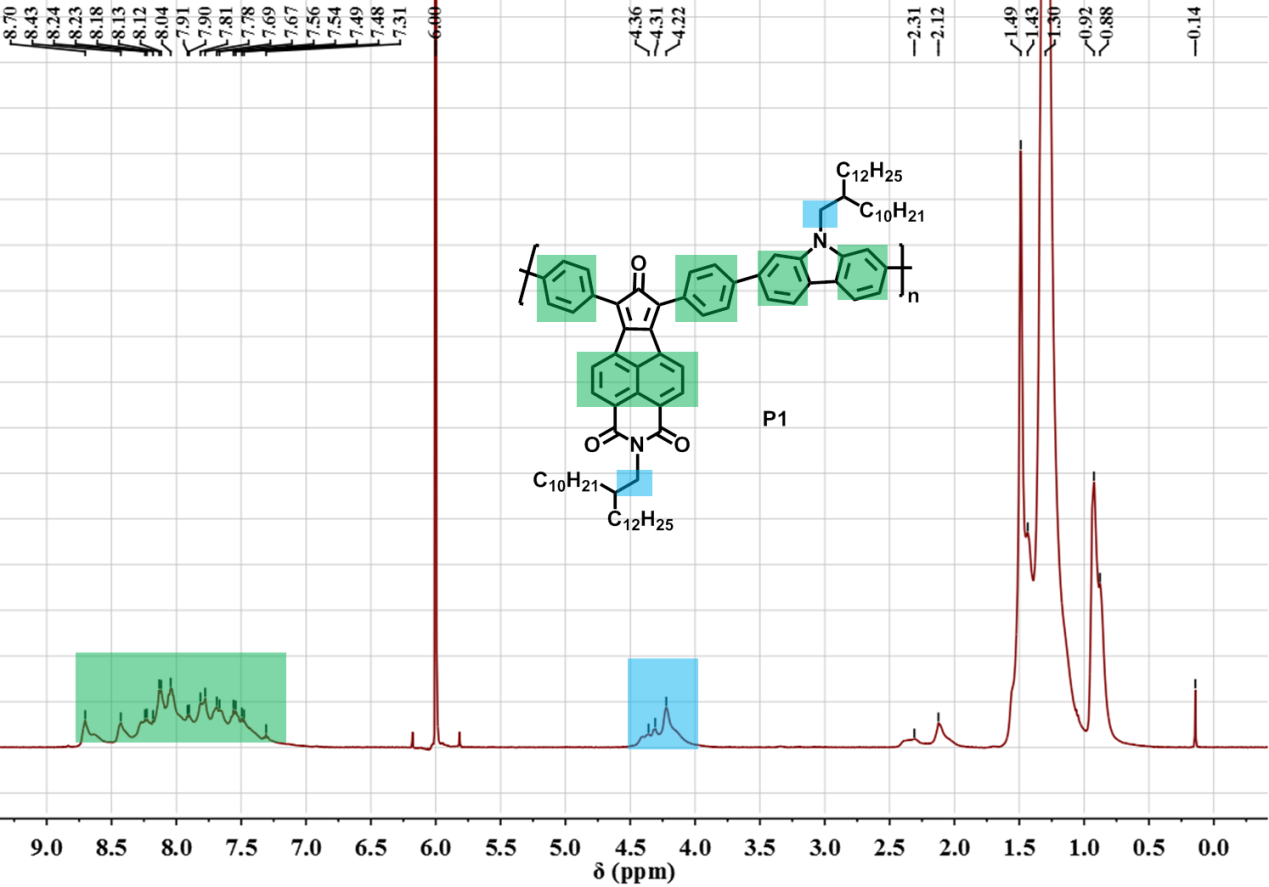
**

**Figure S20.** 500 MHz ^1^H NMR spectrum of **P1** measured in C_2_D_2_Cl_4_ at 373K.


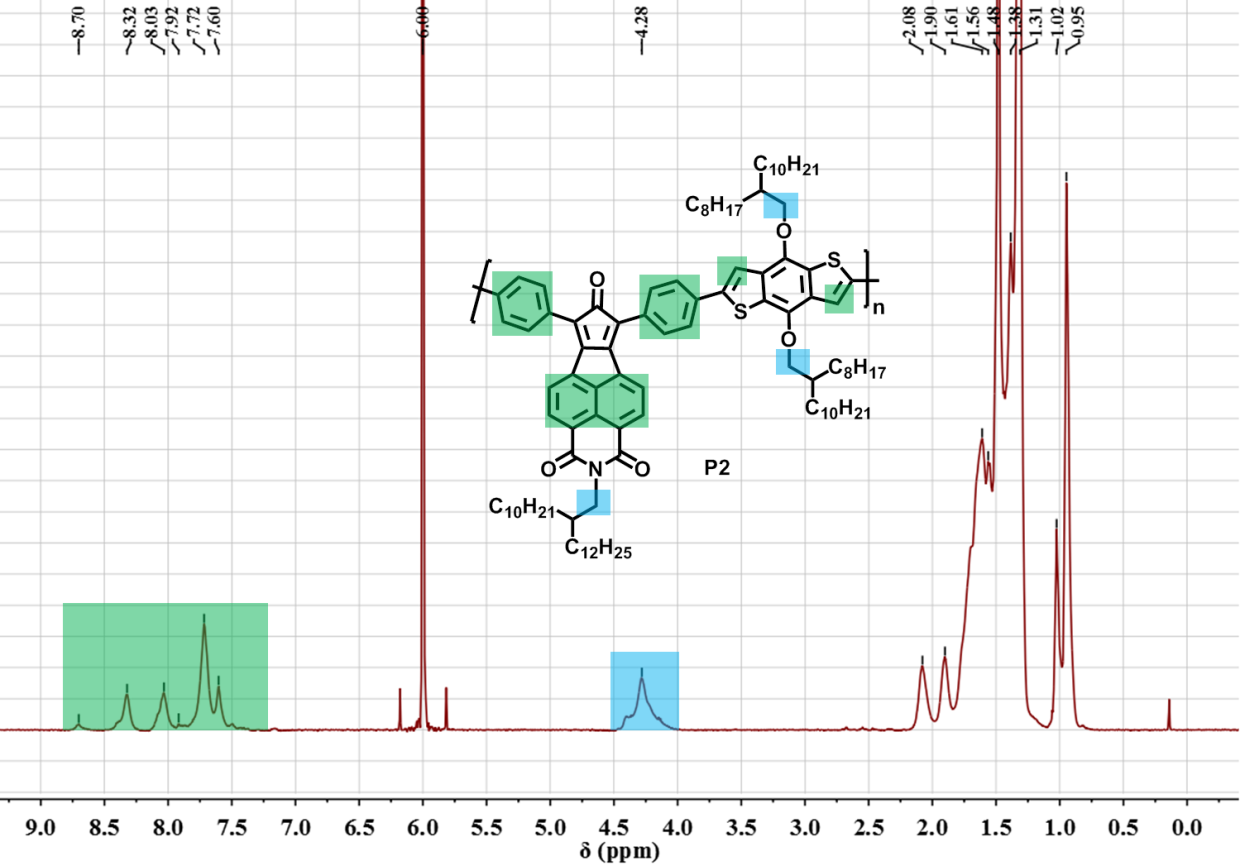


**Figure S21.** 500 MHz ^1^H NMR spectrum of **P2** measured in C_2_D_2_Cl_4_ at 373K.


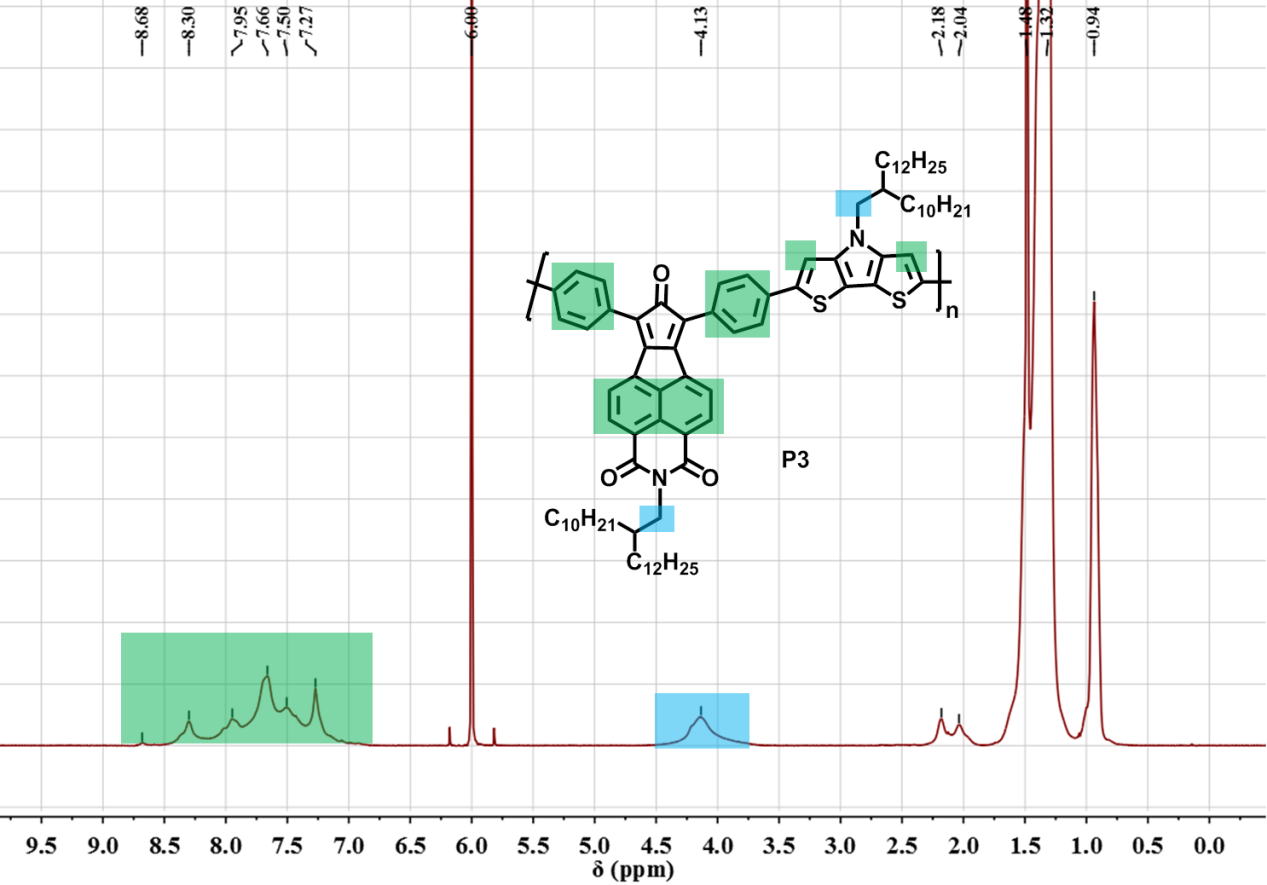


**Figure S22.** 500 MHz ^1^H NMR spectrum of **P3** measured in C_2_D_2_Cl_4_ at 373K.


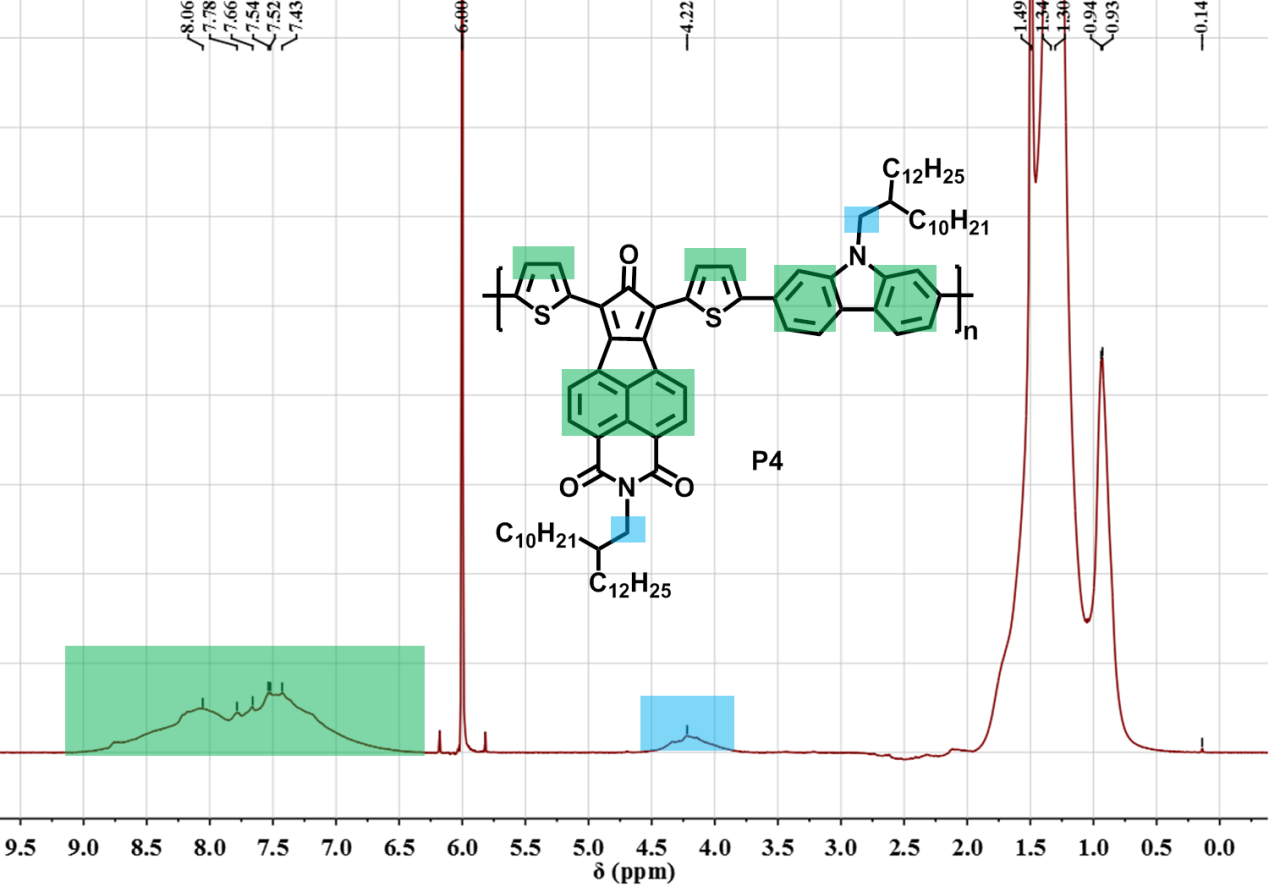


**Figure S23.** 500 MHz ^1^H NMR spectrum of **P4** measured in C_2_D_2_Cl_4_ at 373K.


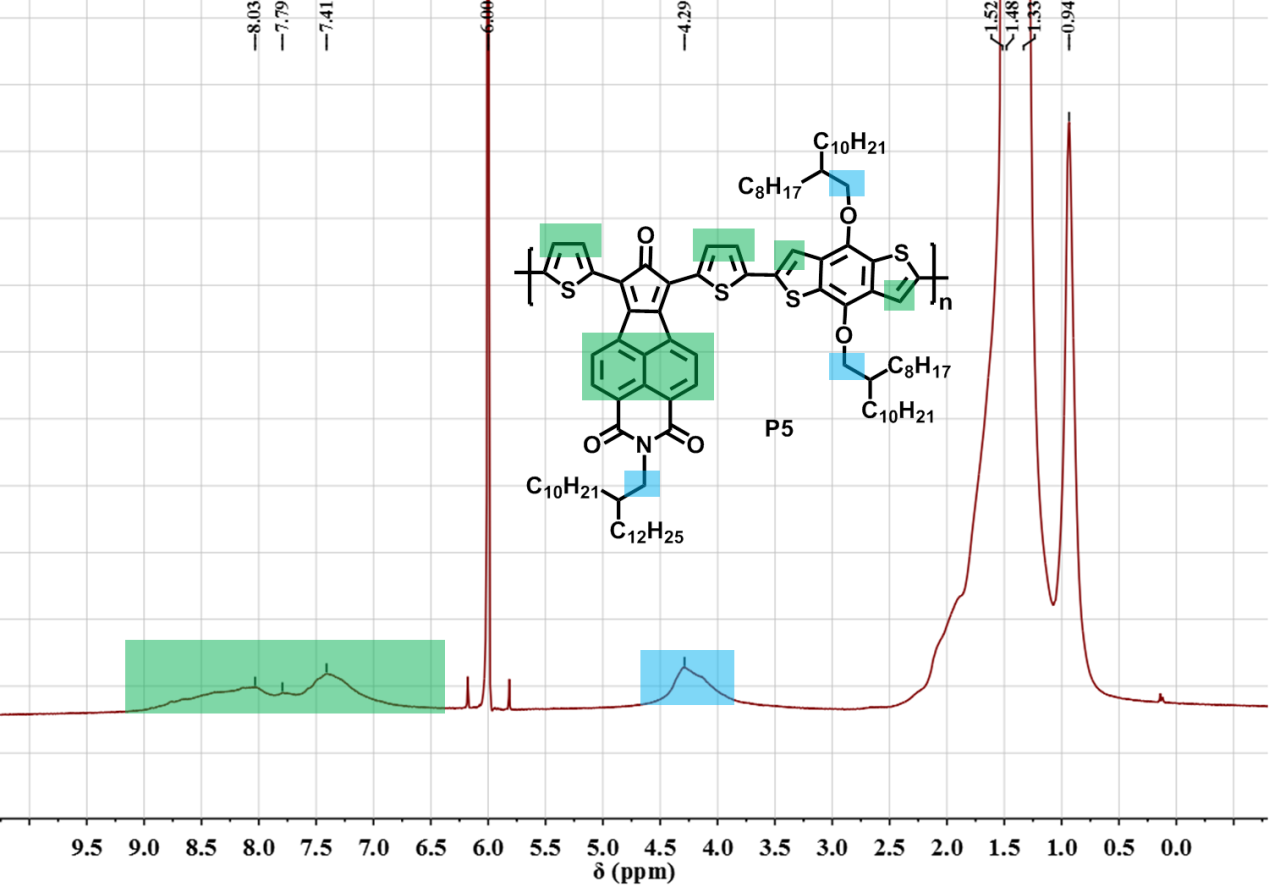


**Figure S24.** 500 MHz ^1^H NMR spectrum of **P5** measured in C_2_D_2_Cl_4_ at 373K.


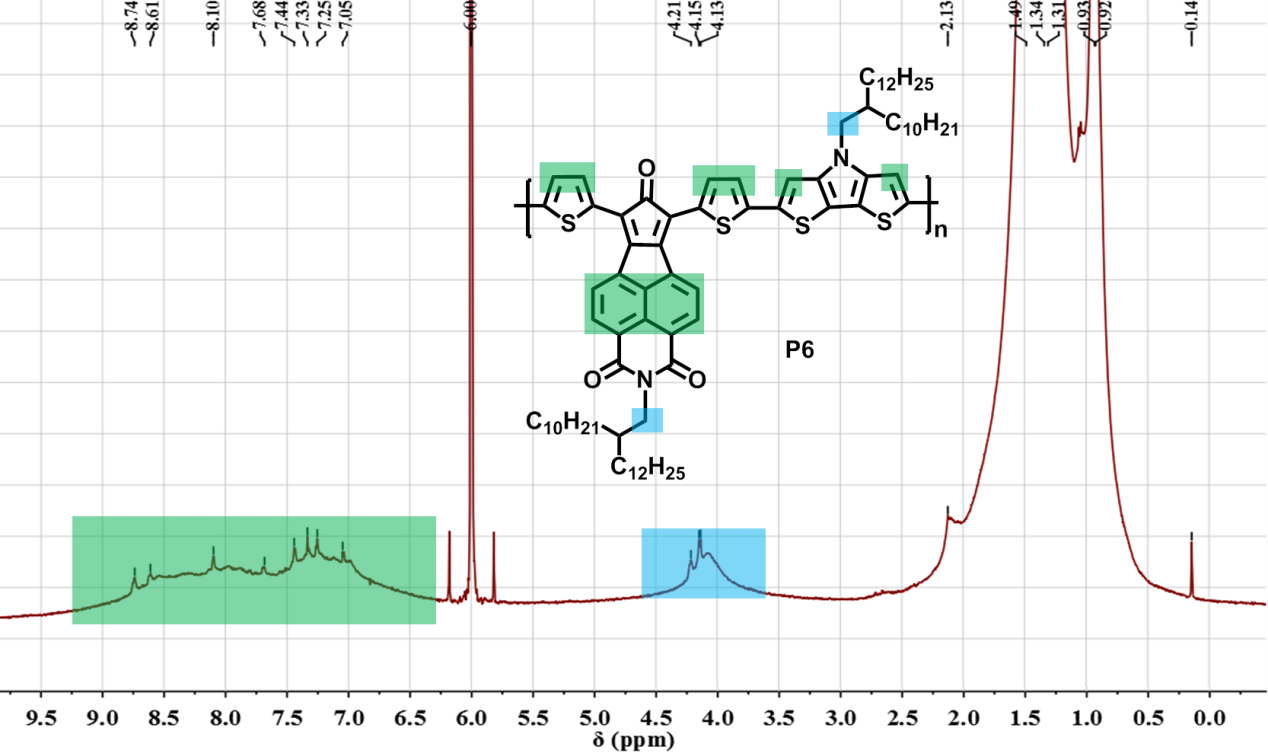


**Figure S25.** 500 MHz ^1^H NMR spectrum of **P6** measured in C_2_D_2_Cl_4_ at 373K.
